# *IGHV1-69* polymorphism modulates anti-influenza antibody repertoires, correlates with IGHV utilization shifts and varies by ethnicity

**DOI:** 10.1038/srep20842

**Published:** 2016-02-16

**Authors:** Yuval Avnir, Corey T. Watson, Jacob Glanville, Eric C. Peterson, Aimee S. Tallarico, Andrew S. Bennett, Kun Qin, Ying Fu, Chiung-Yu Huang, John H. Beigel, Felix Breden, Quan Zhu, Wayne A. Marasco

**Affiliations:** 1 Department of Cancer Immunology & AIDS, Dana-Farber Cancer Institute; Department of Medicine, Harvard Medical School, 450 Brookline Avenue, Boston, Massachusetts 02215, USA; 2 Department of Biological Sciences, Simon Fraser University, Burnaby, British Columbia, V5A 1S6, Canada; 3 Department of Genetics and Genomic Sciences, Icahn School of Medicine at Mount Sinai, New York, NY, USA; 4 Program in Computational and Systems Immunology, Institute for Immunity, Transplantation and Infection, Stanford University School of Medicine, Stanford, California, USA; 5 Division of Biostatistics and Bioinformatics Sidney Kimmel Comprehensive Cancer Center, Johns Hopkins University 550 N. Broadway, Room 1103-A Baltimore, Maryland 21205-2013, USA; 6 Leidos Biomedical Research Inc., Frederick National Laboratory for Cancer Research, Frederick, MD 21702 USA

## Abstract

IGHV polymorphism provides a rich source of humoral immune system diversity. One important example is the *IGHV1-69* germline gene where the biased use of alleles that encode the critical CDR-H2 Phe54 (F-alleles) to make broadly neutralizing antibodies (HV1-69-sBnAb) to the influenza A hemagglutinin stem domain has been clearly established. However, whether *IGHV1-69* polymorphism can also modulate B cell function and Ab repertoire expression through promoter and copy number (CN) variations has not been reported, nor has whether *IGHV1-69* allelic distribution is impacted by ethnicity. Here we studied a cohort of NIH H5N1 vaccinees and demonstrate for the first time the influence of *IGHV1-69* polymorphism on V-segment usage, somatic hypermutation and B cell expansion that elucidates the dominance of F-alleles in HV1-69-sBnAbs. We provide evidence that Phe54/Leu54 (F/L) polymorphism correlates with shifted repertoire usage of other IGHV germline genes. In addition, we analyzed ethnically diverse individuals within the 1000 genomes project and discovered marked variations in F- and L- genotypes and CN among the various ethnic groups that may impact HV1-69-sBnAb responses. These results have immediate implications for understanding HV1-69-sBnAb responses at the individual and population level and for the design and implementation of “universal” influenza vaccine.

Neutralizing antibody (nAb) responses to influenza infection and vaccination are highly variable among individuals throughout the population. This observation can in part be explained by differences associated with health status, exposure history, age and host variability of immune response genes^1^. Since protection is also correlated with nAb titers, any role that immunoglobulin (IG) germline gene polymorphism may play in this variability is important to establish, but has been difficult to investigate due to the use of numerous V, D and J genes in the genesis of immunoglobulins and the enormous combinatorial diversity that results from the pairing of rearranged V_H_ and V_L_ genes. However, the discovery of biased usage of the IG heavy chain variable (IGHV) germline gene *IGHV1-69* in anti-hemagglutinin stem-directed broadly neutralizing Abs (HV1-69-sBnAbs) and the finding that only the heavy chain makes contact with hydrophobic HA stem^2^ has provided a unique opportunity to define the molecular features of anti-influenza BnAbs and simplify immunogenetic studies to understand the contribution of allelic variation at the *IGHV1-69* locus to the anti-influenza sBnAb response.

*IGHV1-69* is one of the most polymorphic loci within the human IGHV gene cluster (14q32.33), exhibiting both allelic and copy number (CN) variation^3^,^4^. There are 14 alleles known to be associated with this gene that can be differentiated by the presence of either a phenylalanine (F) or leucine (L) at amino acid position 54 (Kabat numbering) within the apex of the CDR-H2 loop. Historically, this classification refers to the 51p1-like and hv1263-like allelic groups, respectively (Supplementary Fig. 1a). In addition to coding polymorphisms, the number of *IGHV1-69* germline copies per diploid human genome can vary from 2–4 (Supplementary Fig. 1b)^3^,^5^,^6^, and there are 4 *IGHV1-69* haplotypes with gene duplications in an earlier established American cohort^5^ (Supplementary Fig. 1c).

The relevance of F/L polymorphism to HV1-69-sBnAbs is the fact that almost all of these Abs originate from the *IGHV1-69* F-allelic group. The conserved CDR-H2 Phe54 is a major anchor residue making direct contact with HA, and the replacement of Phe54 by Ala54 or Leu54 (L) has been shown to dramatically reduce binding affinities^7^,^8^. Importantly, in this study and in two recent studies^9^,^10^ the F/L polymorphism is shown to correlate with the frequencies of HV1-69-sBnAbs, being highest in individuals carrying F-alleles. In contrast, the predominant usage of the L-allele group in generation of non-neutralizing anti-gp41 Abs was recently demonstrated in a HIV-1 vaccination study^11^. These recent findings highlight the need to better understand how this genetic variability at the *IGHV1-69* locus can modulate B cell repertoires as well as the extent to which this polymorphism varies across diverse human populations^3^,^5^,^6^,^12^. To address these two questions we analyzed Ab repertoires from an NIH H5N1 vaccinee cohort and samples from the 1000 Genomes Project (1KG)^13^, respectively. We report the new finding that the two allele families have markedly different effects on Ab repertoire expression that is in part explained by CN variation but there are also differences in B cell expansion and somatic hypermutation. In addition, we discovered marked variance in *IGHV1-69* gene duplication and CN among the different ethnic populations that will affect HV1-69-sBnAb responses to influenza vaccines and natural infections.

## Results

### Comparison of antibody responses to H5N1 vaccine among three *IGHV1-69* genotypic groups

**Figure 1:**
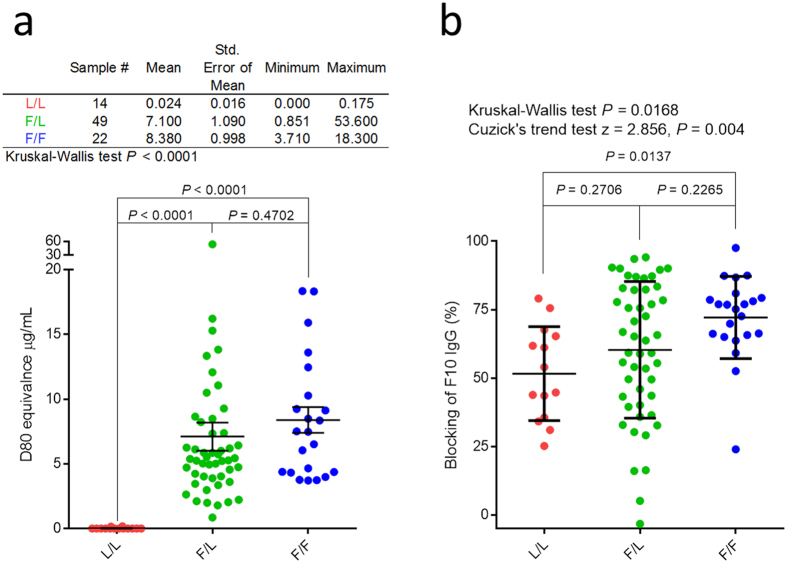
Correlation between *IGHV1-69* polymorphism and Ab response to the H5 vaccine. The pre-vaccinated sera of the 85 individuals were diluted 1/1250 and analyzed for binding activities against the anti-*IGHV1-69* idiotype mAb G6. Binding activities were normalized by subtracting the G6 MSD signal with the MSD signal obtained from an isotype control, and by using a standard curve made with the *IGHV1-69* F-allele-based IgG Ab D80^35^. (**b**) Post-vaccination sera (diluted 1/125) were competed with the anti-stem Ab F10 IgG for binding to H1CA0709. Cuzick’s trend test was used to further confirm that the occurrence of F-alleles increases the ability of serum to block F10 binding (L/L = 0, F/L = 1, F/F = 2). Error bars represent standard error of mean.

Individuals from a 2007 H5N1 vaccination trial were genotyped and phenotyped for *IGHV1-69* CDR-H2 Phe54 F/L polymorphism (rs55891010; see Fig. 1a and **methods**). Their one month post-vaccination sera was competed against the anti-stem sBnAb F10 for binding to the pandemic H1CA0709 HA, which was not circulating when the serum samples were collected. Figure 1b shows a statistically significant difference in F10 blocking activity among the groups and was highest for the F/F group, followed in decreasing order by the F/L and L/L groups. The microneutralization titers (MN) for the F/F group were 1.67 and 2.29 fold higher than the mean values for F/L and L/L groups, respectively with a similar trend in their median values (Supplementary Fig. 2a). The post-vaccination hemagglutination inhibition titers (HAI) and the ELISA titers for H1CA0709 and H1CA0709 HA proteins were shown to not significantly differ from one another among the three *IGHV1-69* genotypic groups (Supplementary Fig. 2b,d,e). In addition, when HAI and MN titers were compared within individuals, there was also a trend toward lower HAI/MN ratios for the F/F and F/L groups compared to the L/L individuals (Supplementary Fig. 2c). Supplementary Fig. 3 shows that stem binding activity originally boosted by H5VN04 vaccination was generally maintained within each genotypic group over the 4-year period. The similar trends observed in the analysis of the F10 competition studies, MN titers, and HAI/MN ratios supports the concept that *IGHV1-69* germline polymorphism has an effect on the profile of the HA-directed Ab response, with expression from F-alleles leading to a higher Ab response to the stem domain.

### Effect on *IGHV1-69* polymorphism on germline gene utilization and expressed HV1-69-sBnAb repertoires

**Figure 2:**
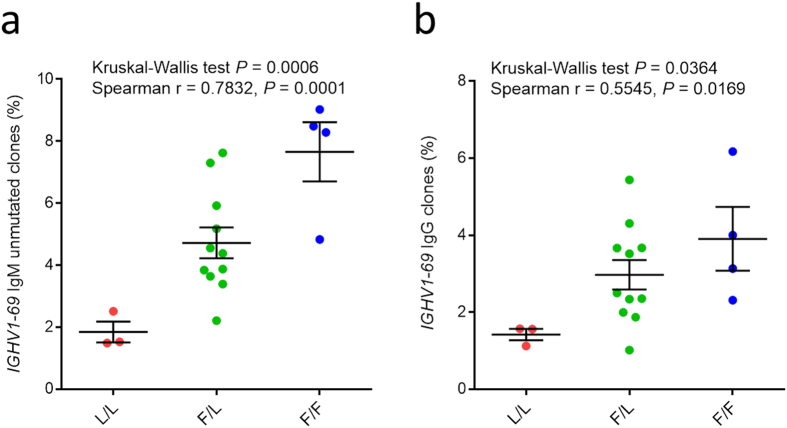
Analyzing *IGHV1-69* V-segment gene utilization among the three *IGHV1-69* genotypic groups. (**a**) The frequency of *IGHV1-69* IgM clones defined by unmutated V-segments (**b**) the frequency of *IGHV1-69* IgG clones. Error bars represent standard error of mean.

To assess the role of IGH locus polymorphism on expressed *IGHV1-69* germline gene repertoires ≥5 × 10^6^ PBMCs (circa 10% B cells) were analyzed from the blood samples of 18 individuals (F/F = 4, F/L = 11, L/L = 3), collected 4 years following the H5N1 vaccine trial. The IGHV-gene frequencies from independent V(D)J rearrangements were rendered non-redundant, and IgM and IgG class determinations were made by analyzing the PCR products obtained from reverse priming with IG constant region primers. Figure 2 shows that in both the unmutated IgM (naïve) and all IgG (memory) V-segment datasets, *IGHV1-69* usage was at the highest frequency in the F/F group (7.7% IgM, 3.9% IgG), intermediate frequency in the F/L group (4.7% IgM, 3% IgG), and the lowest frequency in the L/L group (1.8% IgM, 1.4% IgG). The significance of the ~3-fold difference in *IGHV1-69* usage between the F/F and L/L groups was further demonstrated by noting that, in the F/F group, *IGHV1-69* was the 4^th^ and 7^th^ most frequently used IGHV germline gene in the unmutated IgM and IgG datasets, respectively, whereas in the L/L group *IGHV1-69* was ranked 18^th^ and 23^rd^ (data not shown). This variation in *IGHV1-69* germline gene utilization was also seen for putative HV1-69-sBnAbs with the highest frequencies and correlation coefficients in individuals with F/F alleles and across the IgM B cell subset (Supplementary Fig. 4a–d). We have been able to further delineate some of these HV1-69-sBnAbs signatures through functional analyses (Supplementary Fig. 5 and **text**). These results demonstrate that F-allele individuals have higher levels of circulating *IGHV1-69* Ab and HV1-69-sBnAb repertoires than L-allele individuals.

### Differential effects of *IGHV1-69* genotype on B cell expansion, somatic hypermutation (SHM) and evolution to HV1-69-sBnAb clones

We next investigated if other B cell functions were affected by *IGHV1-69* genotype. Analysis of the naive and memory *IGHV1-69* datasets within each individual’s repertoire revealed additional variation in clonal expansion, SHM frequency, and IgG-to-IgM ratios among each genotypic group. For example, the frequency of highly expanded *IGHV1-69* clones (frequency > 1e-4) was greater for L/L than the F/L or F/F genotypic groups (Supplementary Fig. 6a). However, the clones of the F/F group, of which there were fewer highly expanded clones, were also significantly more mutated than those of the L/L group (Supplementary Fig. 6b). Additionally, we note that *IGHV1-69* is unusual among V-genes in that these BCRs appear at a lower frequency in memory B-cells than in naïve B-cells (Supplementary Fig. 6c) (an approximately 40% reduction)^14^. Interestingly, this effect was strongest in individuals of the F/F genotype. These results suggest that the capacity of the *IGHV1-69* B cells to undergo expansion, SHM and Ig class switching may be different among the genotypic groups.

An expanded dataset of 57 published HV1-69-sBnAbs^2^,^9^,^15^ was also used to investigate the effects of allele variation on SHM and VDJ recombination that results in the signature CDR-H3 amino acids G95, P96 and Y99 ± 1 (Supplementary Fig. 6d). The effects of *IGHV1-69* allelic variation revealed that transition from the germline L54 to the critical F54 in L/L individuals through SHM was a rare event (Supplementary Fig. 6e), as was the occurrence of HV1-69-sBnAb CDR-H3 signatures in the IgG dataset (Supplementary Fig. 6f). The higher frequencies of V-segment amino acid substitutions at positions that are significantly enriched in HV1-69-sBnAbs in the L/L group (Supplementary Fig. 6g) suggests that these Abs may be evolving to compensate for the lack of Phe54^9^. Collectively, this analysis implies that the scarcity of HV1-69-sBnAb in L-allele individuals was due to the underutilization of this allelic type by the immune system.

### *IGHV1-69* Copy Number and Regulatory Region SNPs

**Figure 3:**
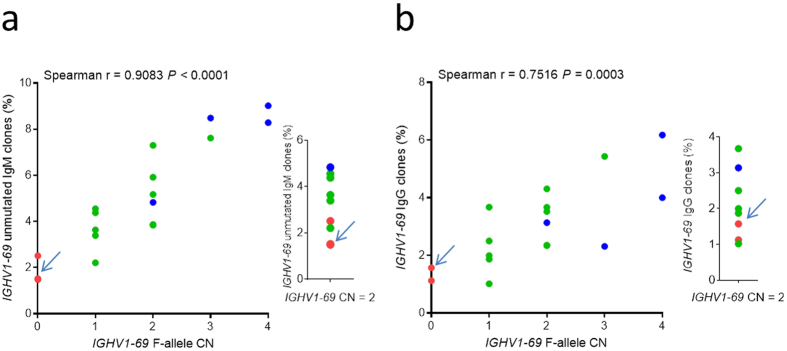
Correlating *IGHV1-69* F-allele copy number with *IGHV1-69* utilization. (**a**) Correlating F-allele CN with the frequency of *IGHV1-69* IgM clones defined by unmutated V-segments. (**b**) Correlating F-allele CN with the frequency of IgG clones. The insets in both panels (**a**) and (**b**) describe *IGHV1-69* clone frequency in individuals that lack *IGHV1-69* gene duplication (Arrows point to overly of two L/L individuals).

We further studied the potential correlation between *IGHV1-69* usage and F-allele copy number (CN)^12^. We found a significant positive correlation between both unmutated IgM and IgG *IGHV1-69* utilization and increasing *IGHV1-69* F-allele copy number (Fig. 3) (Spearman, IgM r = 0.91, *P* < 0.0001; IgG r = 0.75, *P* < 0.0003). A strong positive correlation was also seen between CN and IgM but a weaker one for IgG HV1-69-sBnAb clonal frequencies suggesting that the IgG switch memory B cell subset is subject to additional regulation (Supplementary Fig. 4e,f). Interestingly, all L/L individuals were found to have a mean CN = 2 and they also had the lowest *IGHV1-69* utilization among the three genotypic groups that include individuals whom lack gene duplication (Fig. 3 **insets**), suggesting that CNV only partially explains the lower *IGHV1-69* utilization. For this reason we also investigated other genetic variants in strong linkage disequilibrium (LD) with *IGHV1-69* alleles that could represent SNPs that may influence the control of transcription or V-D-J recombination rates (e.g., variants in the 5′UTR and recombination signal sequences). SNPs within the vicinity of *IGHV1-69* (+− 1.5 kb; GRCh37, chr14:107168431-107171928) in LD with the F/L variant (rs55891010) were identified using data from the 1 KG phase3 dataset for African (n = 661), Asian (n = 504), and European (n = 503) populations. Only four SNPs had an *r*^2^ > 0.8 in at least one of the three populations (Supplementary Fig. 7a). Two of the identified SNPs represented additional coding variants within *IGHV1-69*, and the remaining two occurred upstream of the leader sequence ATG start codon. The SNP rs10220412 was found to reside in the 5′ UTR of *IGHV1-69* and within a promoter initiator element^16^ which is also a binding motif of the B cell associated protein RUNX3, that has been shown to bind to this region in a lymphoblastoid cell line ChIP-seq dataset^17^ (Supplementary Fig. 7b). RUNX3 has been shown to be elevated following EBV infection or activation by PMA of primary B cells and is proposed to have a role in B cell proliferation^18^,^19^. These findings suggest that genetic factors beyond CN can influence *IGHV1-69* transcript frequencies and that the rs10220412 SNP is a candidate that may affect Ab gene transcription in L-allele individuals by hindering the association of RUNX3 to this variant RUNX3/Inr site.

### *IGHV1-69* polymorphism has broad effects on the expressed IGHV repertoire

**Figure 4:**
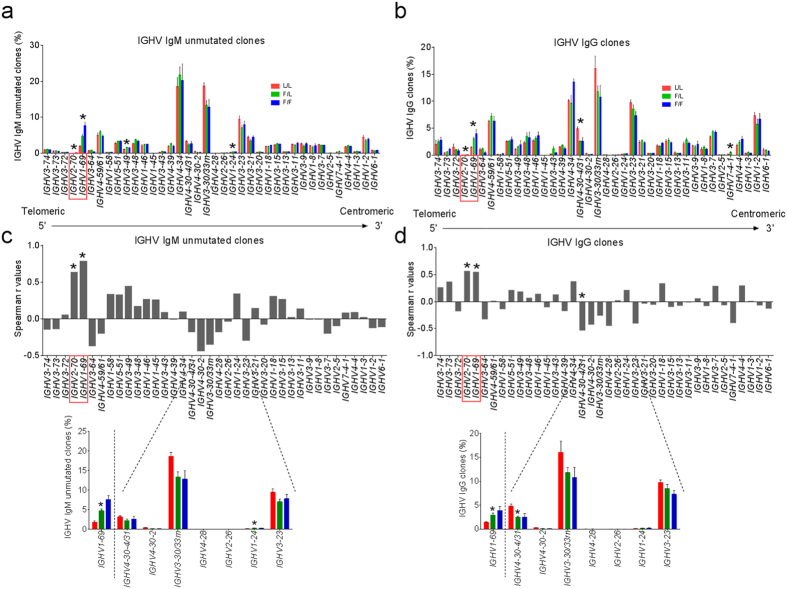
The antibody repertoire of the three *IGHV1-69* genotypic groups. V-gene frequencies were averaged for the L/L group (n = 3), F/L group (n = 11), and F/F group (n = 4) from the datasets of IgM clones characterized by unmutated V-segments (**a**) and IgG clones (**b**). The majority of the functional V-genes were tabulated according to their respective positions in the IGH locus (further detailed in Supplementary Fig. 11). Asterisks denote V-genes utilized differently among the three genotypic groups as determined by Kruskal-Wallis test (*P* < 0.05). Error bars represent standard error of mean. In panels (**c**,**d**) Spearman correlation coefficients are derived for the data presented in panels (**a**,**b**) with L/L = 0, F/L = 1, and F/F = 2. Asterisks indicate statistically significant correlations (*P* < 0.05). Red rectangles point to the location of *IGHV1-69* and *IGHV2-70*, for which their usages were significantly different among the three genotypic groups, being the highest in the F/F group and lowest in the L/L group, in both the unmutated IgM and IgG datasets. The inset panels are enlarged cropped sections from Panel (**a,b**) of the *IGHV4-30-4*/*31*-to-*IGHV3-23* region that is negatively correlated with F-alleles.

The underutilization of *IGHV1-69* germline genes in L/L individuals led us to investigate whether the F/L polymorphism was also associated with different usage frequencies of other V-genes in naïve and memory repertoires. In Fig. 4a,b V-gene frequencies were averaged across individuals within each *IGHV1-69* genotypic group using data from the unmutated IgM and all IgG V-segments, respectively, and aligned according to their relative position in the IGH locus on chr14. In addition to *IGHV1-69, IGHV2-70* utilization was also significantly different in both the unmutated IgM and IgG datasets with repertoire frequencies being highest for the F/F and lowest for the L/L group (Spearman, IgM r = 0.64, *P* = 0.0046; IgG r = 0.57, *P* = 0.0131) (Fig. 4c,d). This is likely explained by the fact that *IGHV1-69* and *IGHV2-70* reside on the same duplicated genomic segment of IGHV, and thus exhibit correlated increases in CN^3^ (Supplementary Fig. 1b). However, we also found evidence of more spatially separated associations between *IGHV1-69* locus polymorphism and other IGHV genes. For example, in the IgG subset, *IGHV4-30-4/31* usage was shown to have a significant positive correlation with the occurrence of L-alleles (r = −0.53, *P* = 0.0223) (**Insert** Fig. 4c (compare red bars)). Although *IGHV4-30-4/31* does not achieve significance in the IgM dataset, it was apparent that *IGHV4-30-4/31* was part of a cluster of IGHV genes which include *IGH4-30-2, IGHV3-30/33rn, IGHV4-28 and IGHV3-23* (**Inset** Fig. 4c,d), all of which were defined by weak to moderate negative correlation coefficients (r = −0.17 to −0.53) and exhibit the highest usage frequencies in L/L individuals. To further assess V-gene usage differences in L/L individuals, we compared V-gene repertoire frequencies between the L/L group and a combined F/L-F/F group using a *t-test*, and visualized these differences using heatmaps (Supplementary Fig. 8a). This analysis revealed that, in the unmutated IgM dataset, *IGHV3-30/33rn* and *IGHV4-30-2* were consistently more highly expressed in the L/L group compared to the F/L-F/F group (*P* < 0.05), whereas *IGHV1-24, IGHV1-69, IGHV2-70* and *IGHV3-49* were significantly underrepresented in L/L individuals (*P* < 0.05). In the IgG subset, *IGHV4-30-2* and *IGHV4-30-4/31* were significantly overrepresented in the L/L group (*P* < 0.05), and again *IGHV1-69* was significantly underrepresented (*P* < 0.05; Supplementary Fig. 8b). Taken together, we observe that multiple clusters of V-genes within the IGHV locus are positively or negatively correlated with *IGHV1-69* genotype.

### *IGHV1-69* F/L polymorphism, copy number and gene duplication among different ethnic groups

**Figure 5:**
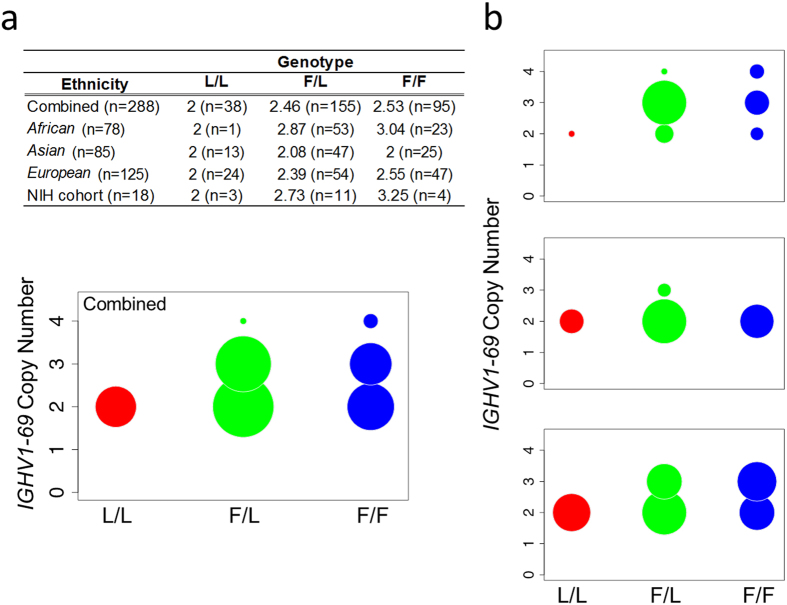
*IGHV1-69* F/L polymorphism and CN variations among various ethnicities. (**a**) Table including mean *IGHV1-69* copy number estimates after partitioning by *IGHV1-69* rs55891010 genotype, provided for the total combined population, for three broad ethnic groups and the NIH cohort samples that were analyzed by NGS. Bubble plots corresponding to *IGHV1- 69* CN for each genotypic class in the total combined population (**a**, lower) and individual ethnic groups (**b**). In each plot, the area of a given circle is proportional to the number of individuals observed for that particular combination of *IGHV1-69* CN and rs55891010 genotype relative to the number of samples analyzed in each group (e.g., Combined, African, Asian, or European).

To further investigate the association between F/L polymorphism and CN we examined published rs55891010 genotypes^13^ and CN^3^ in 288 samples from 3 broad ethnic groups (African, Asian, and European) of the 1 KG Project^13^. Consistent with observations from our H5N1 vaccinee cohort (Fig. 5a **upper table**), in the combined set of 1 KG samples we found a strong association between rs55891010 genotypes and CN, with higher mean *IGHV1-69* CN in F/F (mean = 2.53) and F/L (mean = 2.46) individuals compared to individuals of the L/L genotype (mean = 2; Fig. 5a). We next partitioned these 1 KG samples by ethnicity, which revealed dramatic population differences in frequency of *IGHV1-69* genotypes (Fig. 5a). A significant relationship between *IGHV1-69* genotype and CN was found in Europeans, with mean *IGHV1-69* CN estimates of 2.55, 2.39, and 2, for F/F, F/L, and L/L genotypic classes, respectively. This relationship, however, was not clear in the Asian population, as none of the F/F samples in our analysis were found to have greater than 2 copies of *IGHV1-69* (Fig. 5a,b). Additionally, in the African population, as noted previously^3^, CN was higher on average overall, but in contrast to Europeans, there was a much larger fraction of F/L individuals, and the majority of these samples were found to have 3 copies of *IGHV1-69*. The CN trends were also corroborated by a second *IGHV1-69* gene duplication assay (Supplementary Fig. 9a,b). The African group was also defined by a marked low frequency of L/L individuals (Fig. 5a,b).

Next we expanded our analysis of the *IGHV1-69* rs55891010 polymorphism to include all samples of the 1 KG cohort (Supplementary Fig. 10). We found that the frequency of the L/L genotype varied considerably across human populations, with the lowest frequencies occurring in samples of African ancestry, and the highest in South Asian populations; as expected, opposite trends were noted for the F/F genotype. Taken together, these analyses indicate that interrelationships among *IGHV1-69* F/L genotype, CN and IGHV loci genomic architecture likely exhibit population-specific patterns that may have broad implications for mounting broadly protective HV1-69-sBnAb responses.

## Discussion

There is growing evidence that IG polymorphism may have a critically important role in Ab responses^20^. Allelic variation exists in many IGHV, IGKV, and IGLV germline genes and the results reported herein demonstrate that these genetic differences at the *IGHV1-69* locus can modulate the nAb response. In a cohort of NIH H5N1 vaccinees^21^,^22^, we found higher anti-stem competition and MN titers but not HAI titers in the F/F group. The stem competition results are in agreement with the reports by Pappas^9^ following seasonal influenza vaccination and Wheatley^10^ following a H5 DNA vaccine. Thus, the strong correlation between *IGHV1-69* polymorphism and HV1-69-sBnAb responses provided support for establishing a deeper understanding of the immunobiology of *IGHV1-69* B cells. Likewise, it is important to gain knowledge on the extent to which the distribution of F/F, F/L and L/L individuals vary among the population. Here we demonstrated for the first time the influence of *IGHV1-69* polymorphism on V-segment usage, SHM and B cell expansion that explain the dominance of F-alleles in HV1-69-sBnAbs. We also provided evidence that F/L polymorphism is associated with shifted repertoire usage of other IGHV genes. Marked variations in F- and L- genotypes and *IGHV1-69* CN are also demonstrated among various ethnic groups.

Ab repertoire analysis revealed that *IGHV1-69* utilization increased from L/L to F/L to F/F individuals in both the naive and memory subsets. The correlation observed between F-allele CN and *IGHV1-69* gene usage only partially explained this trend. However the higher *IGHV1-69* gene usage in F-allele individuals whom lack gene duplication over L/L individuals suggested that *IGHV1-69* polymorphism in non-coding regions also had an effect on germline gene expression. We found a strong LD between the F/L polymorphism and SNP rs10220412, located in the 5′ UTR of *IGHV1-69,* within the promoter initiator element^16^ and an annotated binding motif of the B cell associated protein RUNX3^17^. We suggest that the non-consensus mutation in this RUNX3 binding motif found in L/L individuals may affect Ab transcription and/or B cell proliferation^18^,^19^. Additional analyses aimed at investigating the role of SNP rs10220412 will be essential to fully understand the observed relationship between the *IGHV1-69* polymorphism, germline gene utilization and circulating Ab repertoires. Other genetic factors that were not identified in our studies may also be involved.

Our results also show that both *IGHV1-69* F- and L-allele B cells share similar frequency of unmutated IgM clones defined by CDR-H3 HV1-69-sBnAb signatures, however only F-allele B-cells show these elevated signatures in the memory compartment while L-allele B-cells are recessive in their ability to readily resolve HA neutralization in this manner. Indeed, estimates can be made to the frequency of HV1-69-sBnAb precursor B-cells (Supplementary Fig. 6). While 1.8% of the repertoire of an F/F genotype individual and 0.6% of the repertoire of an F/L individual will have an appropriate progenitor CDR-H3 signature rearranged on a *IGHV1-69*-F54 V-gene, an L/L genotype repertoire will have roughly one in ten thousand (0.01%) B-cells configured with the minimal signatures that appear necessary for HV1-69-sBnAbs generation. These results strongly suggest that L/L individuals must reconcile sBnAb responses through the use of other VH germline genes.

The overall variance in *IGHV1-69* utilization among the three genotypic groups was associated with differential usage of other IGHV germline genes. Interestingly, our analyses point to a cluster of IGHV genes circa *IGHV3-30* (Fig. 4: *IGHV4-30-4/31, IGH4-30-2, IGHV3-30/33rn, IGHV4-28 and IGHV3-23*) that are positively correlated with the presence of L-alleles. Although long-range haplotypes spanning the length of the IGHV locus have not been characterized, one possible explanation for this correlation is that L/L individuals could have a distinct IGHV locus architecture that promotes increased VDJ recombination events that preferentially utilize IGHV germline genes within the region of *IGHV3-30.* This could result in shifting of germline gene usage within the Ab repertoire. This positive correlation with L-alleles is of particular interest as there are several reports of sBnAbs that are based on the *IGHV3-30* V-gene^23^,^24^,^25^. Further studies might show that L/L individuals carry additional copies of the *IGHV3-30* and *IGHV3-23* genes, both of which are known to vary in copy number^3^,^20^. Alternatively, it is also plausible that *IGHV1-69* duplication haplotypes contain architectural features that reduce the accessibility and therefore utilization of genes in the *IGHV3-30* region.

Further characterization of CN frequencies in the context of ethnic background using 1 KG samples further supports our observation that gene duplication events (CN > 2) rarely occur in L/L individuals (Fig. 5, Supplementary Fig. 9). However, remarkable population-specific diversity was observed in CN frequencies in the F/L and F/F genotypes. The majority of the African individuals bear *IGHV1-69* gene duplication while in the Asian groups gene duplication hardly occurs. Indeed, nearly every CNV studied in IGHV to date has been shown to exhibit population-specific patterns^3^,^26^,^27^. This remarkable population-specific diversity is also demonstrated with the frequencies of the F/L genotypes. Indeed, the frequency of L/L individuals is miniscule in the African group (~2%), while to the other extreme L/L individuals comprise 41.3% of the South Asian population (Fig. 5, Supplementary Fig. 10). These results imply that individuals of different ethnicities may vary in their capacity to elicit HV1-69-sBnAbs by natural infection or vaccination.

In conclusion, our studies of *IGHV1-69* polymorphism have provided an important entry point to further understand the impact of IGH polymorphism on host nAb responses. We have discovered several important biological associations that had not been previously described. We also uncovered a marked association between *IGHV1-69* polymorphism and ethnicity. The combined genotypic, molecular and phenotypic analyses as presented in this study offer a new approach to understand vaccine responsiveness at the individual and population level. Indeed, *IGHV1-69* genotypes were shown to correlate with anti-HA stem titers and circulating HV1-69-sBnAb repertoires. Clearly defined associations between B cell genotype and phenotype are likely to emerge from these studies, and together with serologic analyses of pre-28,29 and post- exposure Ab responses should lead to important advances in our knowledge of B cell responses to influenza. We propose that the establishment of a complete human IGHV haplotype map is needed to catalogue and map genomic variation in the IGHV locus from larger cohorts of individuals of different ethnicities. This advance will naturally lead to the development of complementary high-throughput genotyping tools that may prove useful for predicting vaccine responsiveness at the individual and population levels. This progress will be particularly important for the development and monitoring of next generation “universal” influenza vaccines^30^,^31^,^32^,^33^,^34^.

## Online Methods

### Ethics statement

All the experiments were performed in accordance with the approved guidelines and regulations of the Institutional Review Boards (IRBs). Specifically, the samples from H5N1 vaccinee cohort at NIH/NIAID were collected under NIH IRB approved Study # 06-I-0235 (Clinical Trials.gov identifier: NCT00383071), titled “A Phase II Vaccine Dose Finding Pilot Study for Development of an Anti-Influenza A (H5Nl) Intravenous Hyper-Immune Globulin”. Informed consent was obtained from all participants. Experiment protocols involving human samples were approved by DFCI IRB and performed under the regulation of the DFCI Legacy # 11–093.

### Details of the H5 vaccination cohort

In the H5N1 vaccination study (Clinical Trials.gov identifier: NCT00383071), the effect of varying amounts of H5 protein and various numbers of vaccinations was tested using the (rgA/H5N1 Vietnam/1203/04 X A/PR/8/34) manufactured by Sanofi Pasteur Inc, Swiftwater, PA. The study concluded that additional vaccinations increased HAI and MN titers, but not increasing vaccine dose. The Kruskal-Wallis test indicated that three groups do not differ in the number of vaccinations (*P* = 0.37), therefore variance in antibody response to the H5N1 vaccine among the three genotypic groups (F/F, F/L, and L/L) is not predicted to be the result of differences number of vaccinations.

### Competing sera with F10 for binding to H1CA0709

MSD 384-well standard plate coated overnight with 6.25 ng of H1CA0709 (A/California/07/2009, Influenza Reagent Resource (IRR) FR-559)) were washed 1 time with PBS and blocked for 1 h at 37 °C with 2% BSA/PBS. After blocking, sera (pre or post) diluted 1/125 in 2% milk/PBST was added to the plate for 45 min at 37 °C, after which 62.5 ng/mL of F10^35^ labeled with sulfo-tag was added for an additional 45 min at 37 °C. Following washes with PBST, read buffer was added and the plate was read using a Sector Imager 2400 instrument. The threshold of 100% inhibition was derived from the wells processed with the dilution buffer, and the threshold of 0% inhibition was obtained from binding of F10 to wells containing dilution buffer.

### MSD ELISA assay for analyzing binding of post-vaccination sera H1CA0709 and H1CA0709 HA1

MSD 384-well high bind plates coated overnight with 25 ng of H1CA0709 and H1CA0709 HA1 (IRR FR-695) were washed 1 time with PBS and blocked for 1 h at 37 °C with 2% BSA/PBS. After blocking, post-vaccination serum samples diluted 1/7500 for H1CA0709 and 1/1500 for H1CA0709 HA1 in 2% milk/PBST were added to the plates for 45 min at 37 °C. Following washes with PBST Goat-anti-human IgG sulfo-tag antibody diluted in 2% milk/PBST was added for 45 min at 37 °C. Following washes with PBST, read buffer was added and the plate was read using a Sector Imager 2400 instrument.

### Next generation sequencing

RNA was extracted from cryopreserved PBMCs by using RiboPure Kit. cDNA was generated by using Superscript RT II kit with 100–600 ng of RNA and oligo-dT primer. VH libraries were generated from cDNA template using Taq polymerase with ThermoPol buffer for multiplex PCR. Primers used for PCR were an equimolar mix of eight forward primers and two reverse primers previously described in^36^. Reactions were carried out using 0.2 μM both forward and reverse primer mixes and 2 μL cDNA in 50 μL total volume. Cycling conditions were as follows: 92 °C denaturation for 3 min, 92 °C for 1 min, 50 °C for 1 m, 72 °C for 1 m for 4 cycles, 92 °C for 1 min, 55 °C for 1 m, 72 °C for 1m for 4 cycles, 92 °C for 1 min, 63 °C for 1 m, 72 °C for 1 m for 20 cycles, 72 °C for 7 min, hold at 4 °C. PCR reactions performed in replicates were pooled and purified by spin column using a QiaQuick PCR Purification Kit. The cDNA products were ligated with indexed Illumina adapters using Thruplex DNAseq. The adapter ligated libraries were quantified by qPCR (Kapa Biosystems cat# KK4824), pooled in equal quantities, and then sequenced using MiSeq 250 bp paired-end reads by the Dana-Farber Cancer Institute Molecular Biology Core Facilities. All kits were used according to the manufacturer’s protocols.

### Genotyping assays

Individuals of the H5N1 vaccination trial were genotyped by real time PCR approach and by ELISA assay with the anti-*IGHV1-69* idiotype mouse mAb G6, which does not bind to *IGHV1-69* Abs displaying CDR-H2 Leu54^37^. This combined genotyping/phenotyping approach enabled us to classify with confidence 85 of the 86 individuals as follows: F/F (G6 + ) = F/L (G6 + ) = 49, and 14 L/L (G6−).

### Real time PCR genotyping approach

Two allelic-group-specific TaqMan (Applied Biosystems) probes were designed to distinguish codon variants of *IGHV1-69*, encoding either the CDR-H2 Leu54 (L-alleles) or Phe54 (F-alleles) alleles, allowing the estimation of the number of copies of each allele in each sample (F-alleles, Forward = TGGACAAGGGCTTGAGTGGAT; Reverse = CCCTGGAACTTCTGTGCGTAGT; Reporter Sequence = CCCTATCTTTGGTACAGC. L-alleles, forward = TGGACAAGGGCTTGAGTGGAT; Reverse = CCCTGGAACTTCTGTGCGTAGT; Reporter Sequence = CCTATCCTTGGTATAGCA, all are 5′-3′). For twenty individuals which donated blood 4 years post vaccination, polymorphonuclear cells were isolated by a standard dextran approach^38^ from which genomic DNA was extracted using QIAGEN’s DNeasy Blood & Tissue Kit. TaqMan PCR assays were performed at least two times with at least three replicates using Applied Biosystems 7300 Real-Time PCR System, and for each sample allelic CN counts were estimated using the delta-delta-CT method. The other 66 individuals were genotyped with the TaqMan probes by using PCR amplified *IGHV1-69* gene product obtained from residual circulating DNA found in the serum. Circulated DNA was isolated from 100–200 μl of pre-vaccination, 1 month post-vaccination, and 4 years post-vaccination sera by using ZR-96 Quick-gDNA Blood kit by ZYMO research, and the *IGHV1-69* gene was amplified using QIAGEN’s HotStarTaq Plus Master Mix Kit, with 3ul of the circulated DNA eluent, and forward primer that anneals in the V-segment intron domain and a reverse primer that anneals in the V-segment RSS domain. Cycling conditions were as follows: 95 °C min, [94 °C 1 min, 50 °C 1 min, 72 °C 1 min] × 50, 72 °C 10 min, 4 °C 16 h. Real time PCR analysis was performed by diluting the PCR products 1-to-1e6 and the TaqMan probes described above were used in order to determine *IGHV1-69* allele composition. Samples were analyzed using CFX384 instrument by Biorad, and Cq and END RFU thresholds for F/F, F/L, and L/L were generated from the sera samples of the 20 individuals which were genotyped using genomic DNA. For samples that did not amplify, a second PCR was performed or residual DNA was isolated again. For confidence purposes both the pre and post-vaccination sera were analyzed, and in situations where there was no agreement residual DNA was isolated again from the pre- and post-vaccination samples and the real time PCR was repeated.

### MSD ELISA assay for analyzing binding of pre-vaccination sera to G6

MSD 384-well high bind plates coated overnight with 6.25 ng of G6 and with isotype control mAb 1D4, were washed 1 time with PBS and blocked for 1 h at 37 °C with 2% BSA/PBS. After blocking pre-vaccination sera diluted 1/1250 in 2% milk/PBST as well as serial dilutions of the *IGHV1-69* F54 allele-based IgG Ab D80^35^ were added to the plate for a period of 1 h at 37 °C. After washing the plates with PBST, Goat-anti-human-IgG sulfo-tag antibody diluted in 2%milk PBST was added for 1 h at 37 °C. Following washes with PBST, read buffer was added and the plate was read using Sector Imager 2400 instrument. G6 binding activities were normalized by subtracting the G6 MSD signal from the MSD signal obtained for 1D4, and by using a standard curve generated from D80 binding activities to G6.

### Analysis of *IGHV1-69* SNP and copy number/duplication data for samples derived from the 1000 Genomes Project

To investigate potential relationships between genotypic variation at the *IGHV1-69* F/L SNP variant (rs55891010) and gene copy number, as well as *IGHV1-69* regulatory polymorphisms, we used available CNV and SNP data from two previous studies^3^,^13^. CNV genotype calls were previously estimated for 425 individuals from a broad set of human populations based on standard PCR and targeted TaqMan qPCR assays unique to sequence characterized within the *IGHV1-69* duplication haplotype (see^3^ and Supplementary Fig. 9a). In total, 288 samples had both CNV and SNP data for comparisons across three broad ethnic groups (African, n = 78; East Asian, n = 85; European, n = 125) represented by 7 subpopulations (Yoruba in Ibadan, Nigeria; Luhya in Webuye, Kenya; Japanese in Tokyo, Japan; Han Chinese in Bejing, China; Toscani in Italy; British in England and Scotland; Finnish in Finland). Additional analyses of allele and genotype frequency differences at rs55891010 in a larger sample of human populations were conducted using genotypes downloaded from the 1 KG project for 5 broad ethnic groups (African, n = 661; East Asian, n = 504; South Asian, n = 489; American, n = 347; European, n = 503).

### Antibody repertoire sequence analysis

Sequences were demultiplexed by Illumina DNA barcodes, converted from fastq to fasta and paired-end assembled. Barcode and primer sequences in the V-segment were then removed from all reads. Sequences were then processed using the AbGenesis VDJFasta pipeline to identify V,D,J segments, isotype, and translated CDRs, as previously described^39^. Samples were rendered clonally non-redundant to include one unique representative of each V + J + CDR-H3 sequence as described in^14^. Somatic hypermutation burden on each clone was determined by number of non-templated base mismatches to the identified V-gene and J-gene, using the closest allele in IMGT^4^.

### Expression of HV1-69-sBnAb precursor clones

The selected six VH genes were synthesized by Genewiz (Plainfield, NJ) with a 5′ SfiI and a 3′ BspEI restriction sites. The VH genes were cloned into two pFarber phagemid scFv display kappa and lambda light chain shuffle libraries and were transformed into electrocompetent TG-1 cells resulting in 1E6-to-1E7 transformants. The phagemid particles were rescued by standard approach utilizing VCSM13 helper phage and phagemid particles were purified by peg-precipitation. Phagemid preps were panned from 1:1 mixed lambda and kappa libraries for H5VN04 (protein sciences (Meriden, CT) by adding 5E12 of phagemid particles. Clone 48.1 was also successfully expressed as scFv-Fc with F10’s light chain using the pcDNA3.4 vector which was transfected into 293T cells using standard polyethylenimine approach.

### MSD assay for bulk phagemids

MSD 384-well high bind plate coated overnight with 25 ng H1CA0709, 25 ng H3PE09 (H3 A/Perth/16/2009, IRR FR-472), 25 ng H5VN04 (H5 A/Vietnam/1203/2004, IRR FR-39), and 25 ng human PDL1 (irrelevant control protein), were washed 1 time with PBS and blocked for 1 h at 37 °C with 2% BSA/PBS. After blocking, peg precipitated bulk phagemid libraries from first round of selection were diluted in 2% milk PBST (1E13 phagemid particles/mL) were added to the wells and plates were incubated at 37 °C for 1 hr. Plates were washed with PBST three times, then sulfo-tagged anti M13 diluted to in 2% milk PBST was added and the plate was incubated for 1 hr at 37 °C and washed again as above. Read buffer was added and plates were read on an MSD Sector Imager.

### Binding kinetic assays

Binding kinetics of 48.1-scFv-Fc was performed using Octet RED (Fortebio, USA) with anti-human Fc sensors. Kinetic cycle consisted of: 1) Equilibrium—sensors were dipped in wells containing kinetic buffer (0.02%/tween, 0.1%/BSA, PBS) for 60 sec. 2) Loading—sensors were dipped in wells containing supernatant of cells transfected with 48.1 (Supplementary Fig. 5c) or 0.05 μg/ml of purified 48.1 scFv for 300 sec (Supplementary Fig. 5d). 3) Baseline—sensors were dipped into wells containing kinetic buffer for 180 sec. 4) Association—sensors were dipped into wells containing 10 μg/ml HA proteins H1CA0709, H2JPN57 (H2 A/Japan/305/1957, IRR FR-700), H5VN04 (protein sciences), H3E09 and H7N3 (H7 A/Netherlands/219/2003, IRR FR-71) (Supplementary Fig. 5c) or serial dilutions of H2JPN57 (Supplementary Fig. 5d) for 300 sec. 5) Dissociation—sensors were dipped into wells containing kinetic buffer for 300 sec (Supplementary Fig. 5c) or 1200 sec (Supplementary Fig. 5d). Association rate (Kon) dissociation rate (kdis) and equilibrium dissociation (KD) constants were calculated using 1:1 fitting model as provided by the Fortebio Data analysis software.

### Statistical analysis

Overall comparisons between the F/F, F/L, and L/L genotype classes were performed by using the Kruskal-Wallis test and subsequent pairwise comparisons were conducted by applying Dunn’s procedure to control the overall Type I error rate. Cuzick’s trend test was used to detect the trend over F/F, F/L, and L/L genotype classes by assigning scores 0, 1, 2 to the three groups; moreover, Spearman’s correlation coefficient was used to summarize the association. Statistical analyses were performed by using Prism 6 (Graphpad Software, Inc.) and R software (www.r-project.org).

## Additional Information

**How to cite this article**: Avnir, Y. *et al. IGHV1-69* polymorphism modulates anti-influenza antibody repertoires, correlates with IGHV utilization shifts and varies by ethnicity. *Sci. Rep.* **6**, 20842; doi: 10.1038/srep20842 (2016).

## Supplementary Material

Supplementary Information

## References

[b1] Franco L. M. . Integrative genomic analysis of the human immune response to influenza vaccination. eLife 2, e00299, doi: 10.7554/eLife.00299 (2013). 23878721 PMC3713456

[b2] Avnir Y. . Molecular signatures of hemagglutinin stem-directed heterosubtypic human neutralizing antibodies against influenza A viruses. PLoS pathogens 10, e1004103, doi: 10.1371/journal.ppat.1004103 (2014). 24788925 PMC4006906

[b3] Watson C. T. . Complete haplotype sequence of the human immunoglobulin heavy-chain variable, diversity, and joining genes and characterization of allelic and copy-number variation. American journal of human genetics 92, 530–546, doi: 10.1016/j.ajhg.2013.03.004 (2013). 23541343 PMC3617388

[b4] Lefranc M. P. Immunoglobulins: 25 years of immunoinformatics and IMGT-ONTOLOGY. Biomolecules 4, 1102–1139, doi: 10.3390/biom4041102 (2014). 25521638 PMC4279172

[b5] Sasso E. H., Willems van Dijk K., Bull A. P. & Milner E. C. A fetally expressed immunoglobulin VH1 gene belongs to a complex set of alleles. The Journal of clinical investigation 91, 2358–2367, doi: 10.1172/JCI116468 (1993). 8099917 PMC443293

[b6] Milner E. C., Hufnagle W. O., Glas A. M., Suzuki I. & Alexander C. Polymorphism and utilization of human VH Genes. Annals of the New York Academy of Sciences 764, 50–61 (1995). 7486575 10.1111/j.1749-6632.1995.tb55806.x

[b7] Lingwood D. . Structural and genetic basis for development of broadly neutralizing influenza antibodies. Nature 489, 566–570, doi: 10.1038/nature11371 (2012). 22932267 PMC7095019

[b8] Throsby M. . Heterosubtypic neutralizing monoclonal antibodies cross-protective against H5N1 and H1N1 recovered from human IgM+ memory B cells. PloS one 3, e3942, doi: 10.1371/journal.pone.0003942 (2008). 19079604 PMC2596486

[b9] Pappas L. . Rapid development of broadly influenza neutralizing antibodies through redundant mutations. Nature 516, 418–422, doi: 10.1038/nature13764 (2014). 25296253

[b10] Wheatley A. K. . H5N1 Vaccine-Elicited Memory B Cells Are Genetically Constrained by the IGHV Locus in the Recognition of a Neutralizing Epitope in the Hemagglutinin Stem. Journal of immunology 195, 602–610, doi: 10.4049/jimmunol.1402835 (2015). PMC4491024 26078272

[b11] Williams W. B. . Diversion of HIV-1 vaccine-induced immunity by gp41-microbiota cross-reactive antibodies. Science, doi: 10.1126/science.aab1253 (2015). PMC4562404 26229114

[b12] Sasso E. H., Johnson T. & Kipps T. J. Expression of the immunoglobulin VH gene 51p1 is proportional to its germline gene copy number. The Journal of clinical investigation 97, 2074–2080, doi: 10.1172/JCI118644 (1996). 8621797 PMC507282

[b13] Abecasis G. R. . A map of human genome variation from population-scale sequencing. Nature 467, 1061–1073, doi: 10.1038/nature09534 (2010). 20981092 PMC3042601

[b14] Glanville J. . Naive antibody gene-segment frequencies are heritable and unaltered by chronic lymphocyte ablation. Proceedings of the National Academy of Sciences of the United States of America. 108, 20066–20071, doi: 10.1073/pnas.1107498108 (2011). 22123975 PMC3250199

[b15] Whittle J. R. . Flow cytometry reveals that H5N1 vaccination elicits cross-reactive stem-directed antibodies from multiple Ig heavy-chain lineages. Journal of virology. 88, 4047–4057, doi: 10.1128/JVI.03422-13 (2014). 24501410 PMC3993745

[b16] Roy A. L., Sen R. & Roeder R. G. Enhancer-promoter communication and transcriptional regulation of Igh. Trends in immunology 32, 532–539, doi: 10.1016/j.it.2011.06.012 (2011). 21855411 PMC3200469

[b17] Harrow J. . GENCODE: the reference human genome annotation for The ENCODE Project. Genome research 22, 1760–1774, doi: 10.1101/gr.135350.111 (2012). 22955987 PMC3431492

[b18] Spender L. C., Cornish G. H., Sullivan A. & Farrell P. J. Expression of transcription factor AML-2 (RUNX3, CBF(alpha)-3) is induced by Epstein-Barr virus EBNA-2 and correlates with the B-cell activation phenotype. Journal of virology 76, 4919–4927 (2002). 11967309 10.1128/JVI.76.10.4919-4927.2002 PMC136164

[b19] Brady G. & Farrell P. J. RUNX3-mediated repression of RUNX1 in B cells. Journal of cellular physiology 221, 283–287, doi: 10.1002/jcp.21880 (2009). 19603429

[b20] Watson C. T. & Breden F. The immunoglobulin heavy chain locus: genetic variation, missing data, and implications for human disease. Genes and immunity 13, 363–373, doi: 10.1038/gene.2012.12 (2012). 22551722

[b21] Sui J. . Wide prevalence of heterosubtypic broadly neutralizing human anti-influenza A antibodies. Clinical infectious diseases : an official publication of the Infectious Diseases Society of America 52, 1003–1009, doi: 10.1093/cid/cir121 (2011). 21460314 PMC3070035

[b22] Beige J. H., Voell J., Huang C. Y., Burbelo P. D. & Lane H. C. Safety and immunogenicity of multiple and higher doses of an inactivated influenza A/H5N1 vaccine. The Journal of infectious diseases. 200, 501–509, doi: 10.1086/599992 (2009). 19569973 PMC3417327

[b23] Wyrzucki A. . Alternative recognition of the conserved stem epitope in influenza A virus hemagglutinin by a VH3-30-encoded heterosubtypic antibody. Journal of virology. 88, 7083–7092, doi: 10.1128/JVI.00178-14 (2014). 24719426 PMC4054347

[b24] Corti D. . A neutralizing antibody selected from plasma cells that binds to group 1 and group 2 influenza A hemagglutinins. Science 333, 850–856, doi: 10.1126/science.1205669 (2011). 21798894

[b25] Nakamura G. . An *in vivo* human-plasmablast enrichment technique allows rapid identification of therapeutic influenza A antibodies. Cell host & microbe 14, 93–103, doi: 10.1016/j.chom.2013.06.004 (2013). 23870317

[b26] Sasso E. H., Buckner J. H. & Suzuki L. A. Ethnic differences in VH gene polymorphism. Annals of the New York Academy of Sciences 764, 72–73 (1995). 7486592 10.1111/j.1749-6632.1995.tb55808.x

[b27] Shin E. K. . Polymorphism of the human immunoglobulin variable region segment V1-4.1. Immunogenetics. 38, 304–306 (1993). 8319982 10.1007/BF00188810

[b28] Andrews S. F. . High preexisting serological antibody levels correlate with diversification of the influenza vaccine response. Journal of virology 89, 3308–3317, doi: 10.1128/JVI.02871-14 (2015). 25589639 PMC4337521

[b29] Henry Dunand C. J. . Preexisting human antibodies neutralize recently emerged H7N9 influenza strains. The Journal of clinical investigation 125, 1255–1268, doi: 10.1172/JCI74374 (2015). 25689254 PMC4362269

[b30] Krammer F. & Palese P. Universal influenza virus vaccines: need for clinical trials. Nature immunology. 15, 3–5, doi: 10.1038/ni.2761 (2014). 24352315

[b31] Krammer F., Pica N., Hai R., Margine I. & Palese P. Chimeric hemagglutinin influenza virus vaccine constructs elicit broadly protective stalk-specific antibodies. Journal of virology 87, 6542–6550, doi: 10.1128/JVI.00641-13 (2013). 23576508 PMC3676110

[b32] Wohlbold T. J. . Vaccination with soluble headless hemagglutinin protects mice from challenge with divergent influenza viruses. Vaccine 33, 3314–3321, doi: 10.1016/j.vaccine.2015.05.038 (2015). 26026378 PMC4472732

[b33] Yassine H. M. . Hemagglutinin-stem nanoparticles generate heterosubtypic influenza protection. Nature medicine 21, 1065–1070, doi: 10.1038/nm.3927 (2015). 26301691

[b34] Impagliazzo A. . A stable trimeric influenza hemagglutinin stem as a broadly protective immunogen. Science, doi: 10.1126/science.aac7263 (2015). 26303961

[b35] Sui J. . Structural and functional bases for broad-spectrum neutralization of avian and human influenza A viruses. Nature structural & molecular biology 16, 265–273, doi: 10.1038/nsmb.1566 (2009). PMC2692245 19234466

[b36] Ippolito G. C. . Antibody repertoires in humanized NOD-scid-IL2Rgamma(null) mice and human B cells reveals human-like diversification and tolerance checkpoints in the mouse. PloS one 7, e35497, doi: 10.1371/journal.pone.0035497 (2012). 22558161 PMC3338711

[b37] Potter K. N., Li Y., Mageed R. A., Jefferis R. & Capra J. D. Molecular characterization of the VH1-specific variable region determinants recognized by anti-idiotypic monoclonal antibodies G6 and G8. Scandinavian journal of immunology 50, 14–20 (1999). 10404046 10.1046/j.1365-3083.1999.00524.x

[b38] Clark R. A. & Nauseef W. M. In Current Protocols in Immunology (John Wiley & Sons, Inc., 2001).

[b39] Glanville J. . Precise determination of the diversity of a combinatorial antibody library gives insight into the human immunoglobulin repertoire. Proceedings of the National Academy of Sciences of the United States of America 106, 20216–20221, doi: 10.1073/pnas.0909775106 (2009). 19875695 PMC2787155

