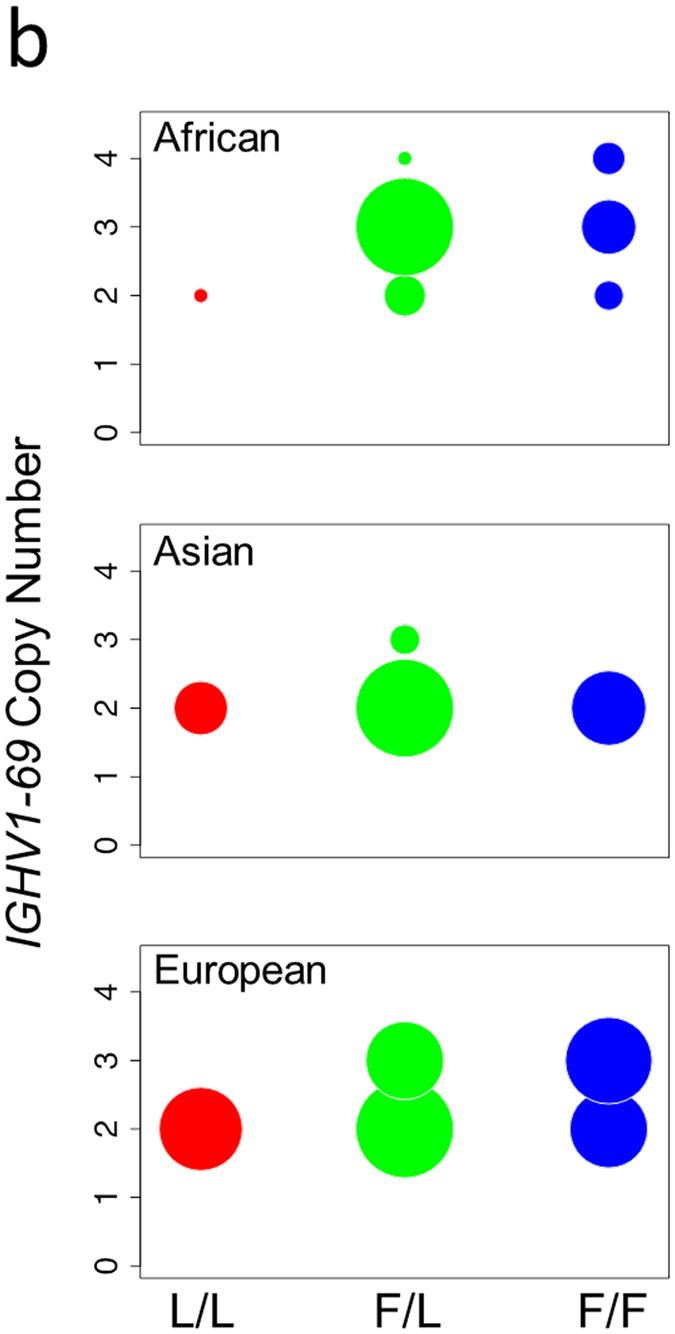# Erratum: *IGHV1-69* polymorphism modulates anti-influenza antibody repertoires, correlates with IGHV utilization shifts and varies by ethnicity

**DOI:** 10.1038/srep23876

**Published:** 2016-04-29

**Authors:** Yuval Avnir, Corey T. Watson, Jacob Glanville, Eric C. Peterson, Aimee S. Tallarico, Andrew S. Bennett, Kun Qin, Ying Fu, Chiung-Yu Huang, John H. Beigel, Felix Breden, Quan Zhu, Wayne A. Marasco

Scientific Reports 6: Article number: 20842; 10.1038/srep20842 published online: 02162016; updated: 04292016.

In this Article, the Figure labels were omitted from Figure 5b. The correct Figure 5b appears below as Fig. 1.

## Figures and Tables

**Figure 1:**