# Supplementary material for: IGHV1-69 polymorphism modulates anti-influenza antibody repertoires, correlates with IGHV utilization shifts and varies by ethnicity
Source: Sci Rep. 2016 Feb 16;6:20842. doi: 10.1038/srep20842 (PMC4754645; doi:10.1038/srep20842)
Supplement: Supplementary Information [file srep20842-s1.pdf]

IGHV1-69 polymorphism modulates anti-influenza antibody repertoires, correlates with IGHV utilization shifts and varies by ethnicity

Yuval Avnir[1], Corey T. Watson[2,3], Jacob Glanville[4], Eric C. Peterson[1], Aimee S. Tallarico[1], Andrew S. Bennett[1], Kun Qin[1], Ying Fu[1], Chiung-Yu Huang[5], John H. Beigel[6], Felix Breden[2], Quan Zhu[1], and Wayne A. Marasco*[1].

[1] - Department of Cancer Immunology & AIDS, Dana-Farber Cancer Institute; Department of Medicine, Harvard Medical School, 450 Brookline Avenue, Boston, Massachusetts 02215, USA.

[2] - Department of Biological Sciences, Simon Fraser University, Burnaby, British Columbia, V5A 1S6, Canada.

[3] - Department of Genetics and Genomic Sciences, Icahn School of Medicine at Mount Sinai, New York, NY, USA.

[4] - Program in Computational and Systems Immunology, Institute for Immunity, Transplantation and Infection, Stanford University School of Medicine, Stanford, California, USA.

[5] - Division of Biostatistics and Bioinformatics Sidney Kimmel Comprehensive Cancer Center, Johns Hopkins University 550 N. Broadway, Room 1103-A Baltimore, Maryland 21205-2013, USA.

[6] - Leidos Biomedical Research Inc., Frederick National Laboratory for Cancer Research, Frederick, MD 21702 USA.

*** Corresponding Author:**
Wayne A. Marasco, M.D., Ph.D.
Department of Cancer Immunology & AIDS
Dana-Farber Cancer Institute
Harvard Medical School
450 Brookline Ave. JF 824
Boston, MA02215
Office (617) 632-2153
Fax (617) 632-3889
Email: wayne_marasco@dfci.harvard.edu.

Figure S1

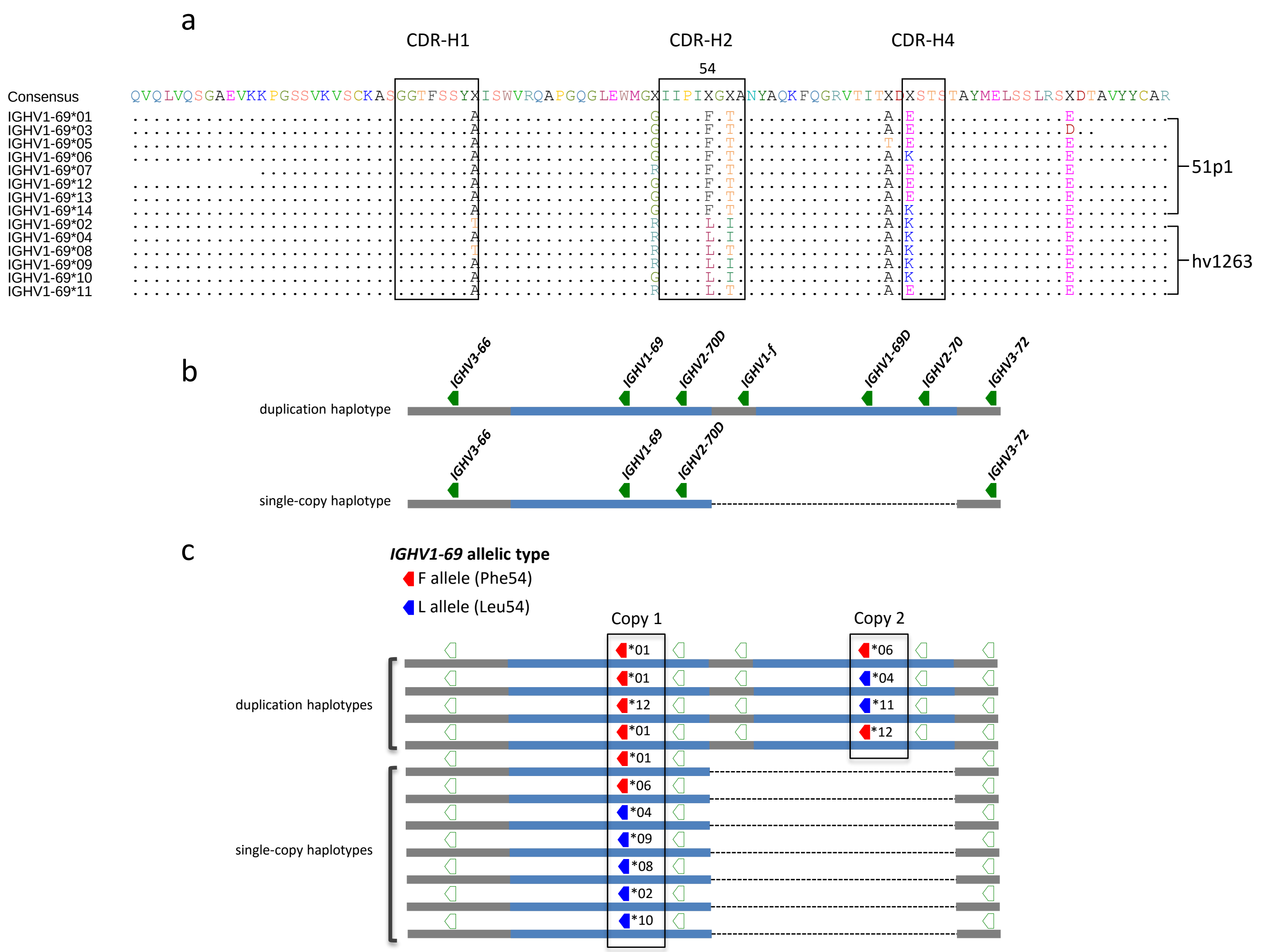
a
CDR-H1
CDR-H2
CDR-H4
54
Consensus
IGHV1-69*01
IGHV1-69*03
IGHV1-69*05
IGHV1-69*06
IGHV1-69*07
IGHV1-69*12
IGHV1-69*13
IGHV1-69*14
IGHV1-69*02
IGHV1-69*04
IGHV1-69*08
IGHV1-69*09
IGHV1-69*10
IGHV1-69*11
51p1
hv1263
b
duplication haplotype
single-copy haplotype
IGHV3-66
IGHV1-69
IGHV2-70D
IGHV1-f
IGHV1-69D
IGHV2-70
IGHV3-72
c
IGHV1-69 allelic type
F allele (Phe54)
L allele (Leu54)
Copy 1
Copy 2
duplication haplotypes
single-copy haplotypes

Supplementary Figure 1. *IGHV1-69* is highly polymorphic with respect to allelic and haplotype structural variation.

(**a**) Consensus alignment of the *IGHV1-69* allele amino acid sequences. The amino acid sequences of *IGHV1-69* alleles were aligned and ordered based on the occurrence of Phe (51p1) or Leu (hv1263) in position 54 (Kabat numbering). Boxes highlight the location of CDR-H1 and CDR-H2 (IMGT, the international ImMunoGeneTics information system®[1]) and of CDR-H4[2]. Positions where there is not 100% match among the alleles are depicted with colored amino acids. (**b**) Schematic representation of two known human *IGHV1-69* structural haplotypes characterized at nucleotide resolution; one haplotype harbors only a single copy of *IGHV1-69* and the neighboring gene *IGHV2-70* (lower), whereas a second more complex duplication haplotype harbors two unique copies of both *IGHV1-69* and *IGHV2-70*, and a single copy of *IGHV1-f*[3]. In the context of these fully sequenced haplotypes, panel (**c**) depicts 11/12 human haplotypes originally characterized in an American cohort of 48 individuals[4,5] using restriction fragment length polymorphism (RFLP). Both copy number and allelic information are shown, including whether *IGHV1-69* gene copies are of the F (Phe54) or L (Leu54) type, as indicated by either red or blue chevrons, respectively. Allele names, as catalogued in IMGT, are indicated for each *IGHV1-69* copy on the 11 haplotypes; although 12 haplotypes were originally reported[4,5] based on RFLP profiles, two of these could not be differentiated based solely on *IGHV1-69* IMGT alleles, and were thus collapsed into one haplotype in the present figure.

Figure S2

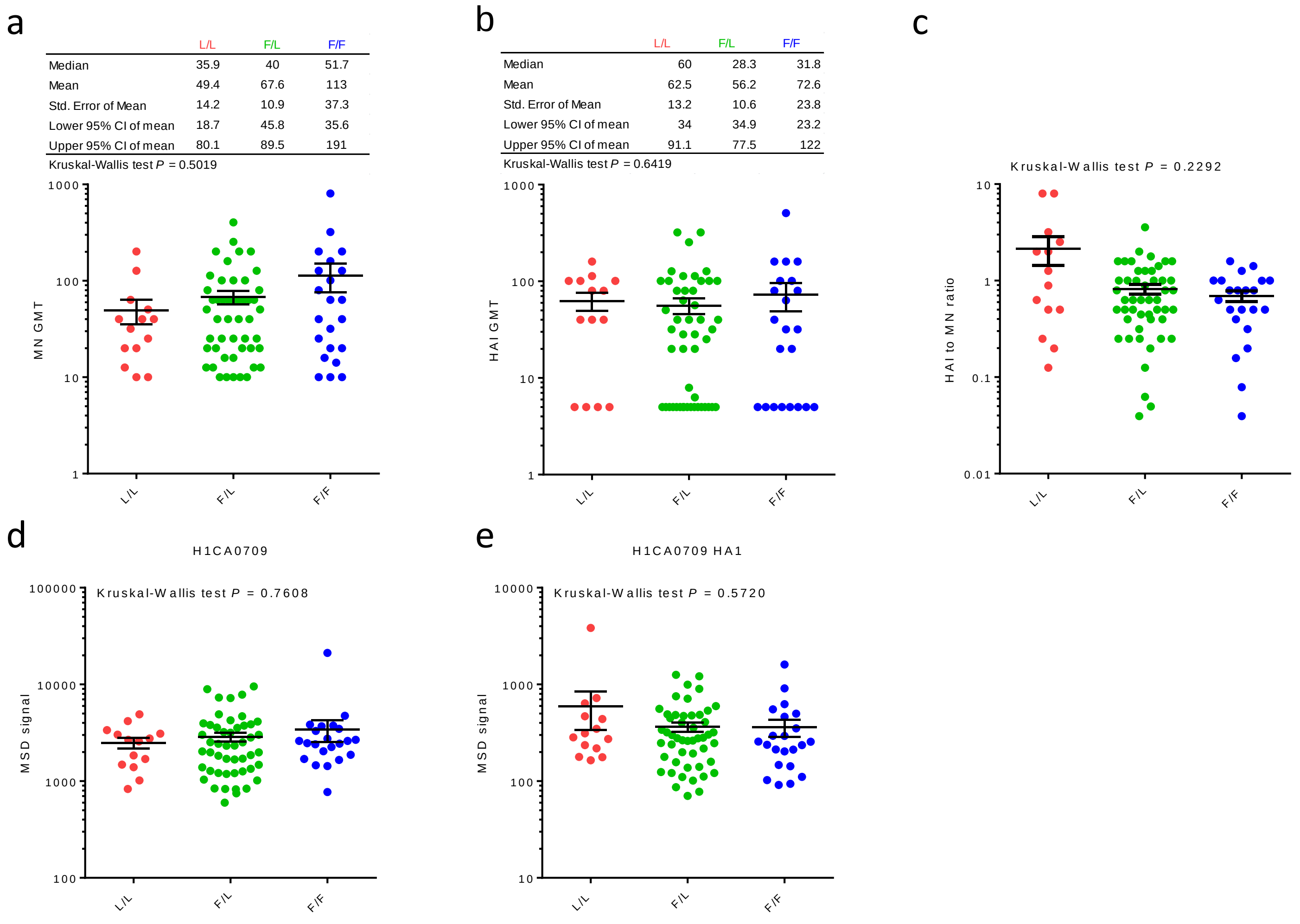

Supplementary Figure 2. Correlation between *IGHV1-69* polymorphism and Ab response to the H5 vaccine.

The post-vaccination MN (**a**) and HAI (**b**) titers were compared among the three *IGHV1-69* genotypic groups. MN titer background level was set to the value of 10 and HAI background level was set to the value of 5. (**c**) HAI titers of each individual were divided by their respective MN titers. (**d**) Binding activities of the post-vaccination sera to full length H1CA0709 (diluted 1/7500) and to (**e**) the HA1 chain of H1CA0709 (diluted 1/1500). Kruskal-Wallis test was used to test the overall difference among the genotypic groups. Error bars represent standard error of mean.

Figure S3

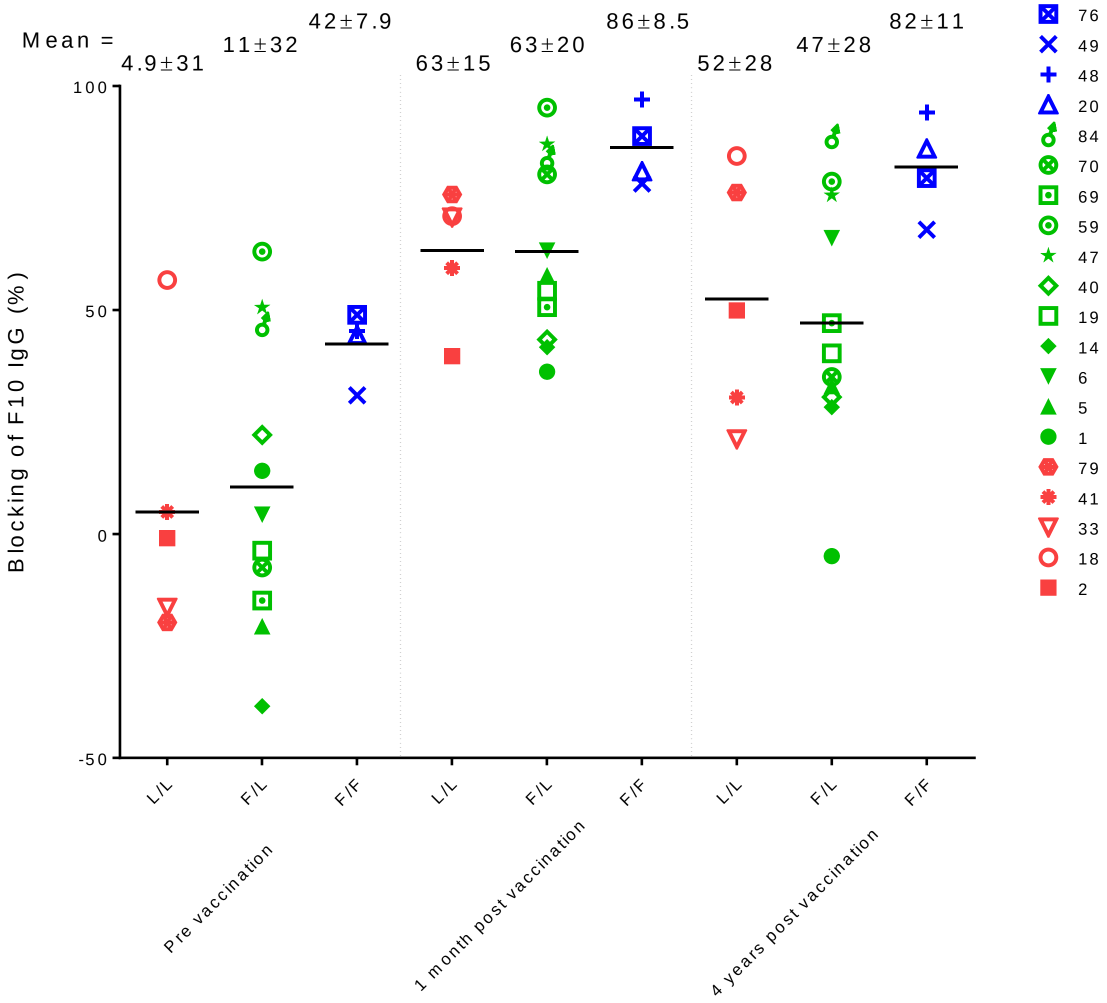

Supplementary Figure 3. Analyzing the capabilities of sera obtained from pre-vaccination, post-vaccination and 4 years post-vaccination to block anti-stem Ab from binding to H1CA0709.

The pre-vaccination, 1-to-4 months post vaccinated, and 4 year post-vaccination sera (diluted 1/125) were competed with the anti-stem Ab F10 IgG for binding to H1CA0709. While a boosting effect by post-2007 seasonal influenza vaccinations cannot be ruled out, the trend in blocking activities is maintained 4 years post-vaccination among the three genotypic groups, with the F/F group being the highest. Error bars represent standard error of mean.

Figure S4

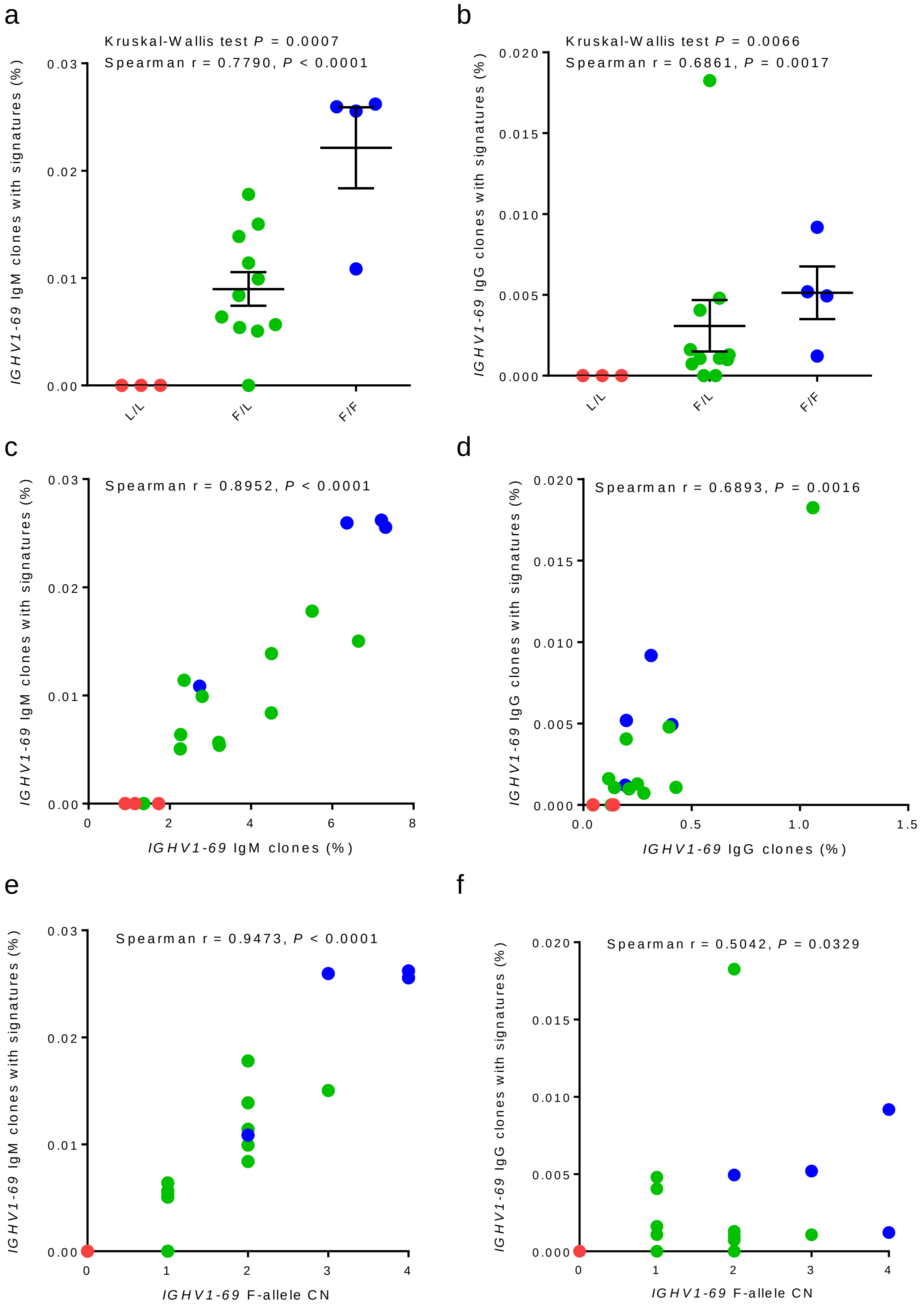
a
Kruskal-Wallis test P = 0.0007
Spearman r = 0.7790, P < 0.0001
IGHV1-69 IgM clones with signatures (%)
0.03
0.02
0.01
0.00
L/L
F/L
F/F
b
Kruskal-Wallis test P = 0.0066
Spearman r = 0.6861, P = 0.0017
IGHV1-69 IgG clones with signatures (%)
0.020
0.015
0.010
0.005
0.000
L/L
F/L
F/F
c
Spearman r = 0.8952, P < 0.0001
IGHV1-69 IgM clones with signatures (%)
IGHV1-69 IgM clones (%)
0
2
4
6
8
d
Spearman r = 0.6893, P = 0.0016
IGHV1-69 IgG clones with signatures (%)
IGHV1-69 IgG clones (%)
0.0
0.5
1.0
1.5
e
Spearman r = 0.9473, P < 0.0001
IGHV1-69 IgM clones with signatures (%)
IGHV1-69 F-allele CN
f
Spearman r = 0.5042, P = 0.0329
IGHV1-69 IgG clones with signatures (%)
IGHV1-69 F-allele CN

Supplementary Fig. 4. Studying correlations between the frequency of HV1-69-sBnAb precursor clones, *IGHV1-69* clones and *IGHV1-69* F-allele copy number.

The IgM and IgG unique clone datasets were analyzed for the frequency of IgM (**a**) and IgG (**b**) HV1-69-sBnAb precursor clones[2,6-8] among the three genotypic groups and were correlated with the respective *IGHV1-69* IgM (**c**) and IgG (**d**) clone frequencies and with *IGHV1-69* F-allele copy number (**e** and **f**). Kruskal-Wallis test was used to test the overall difference among the genotypic groups and Spearman's correlation coefficient (r) was used to summarize the correlation. Error bars represent standard error of mean.

Figure S5

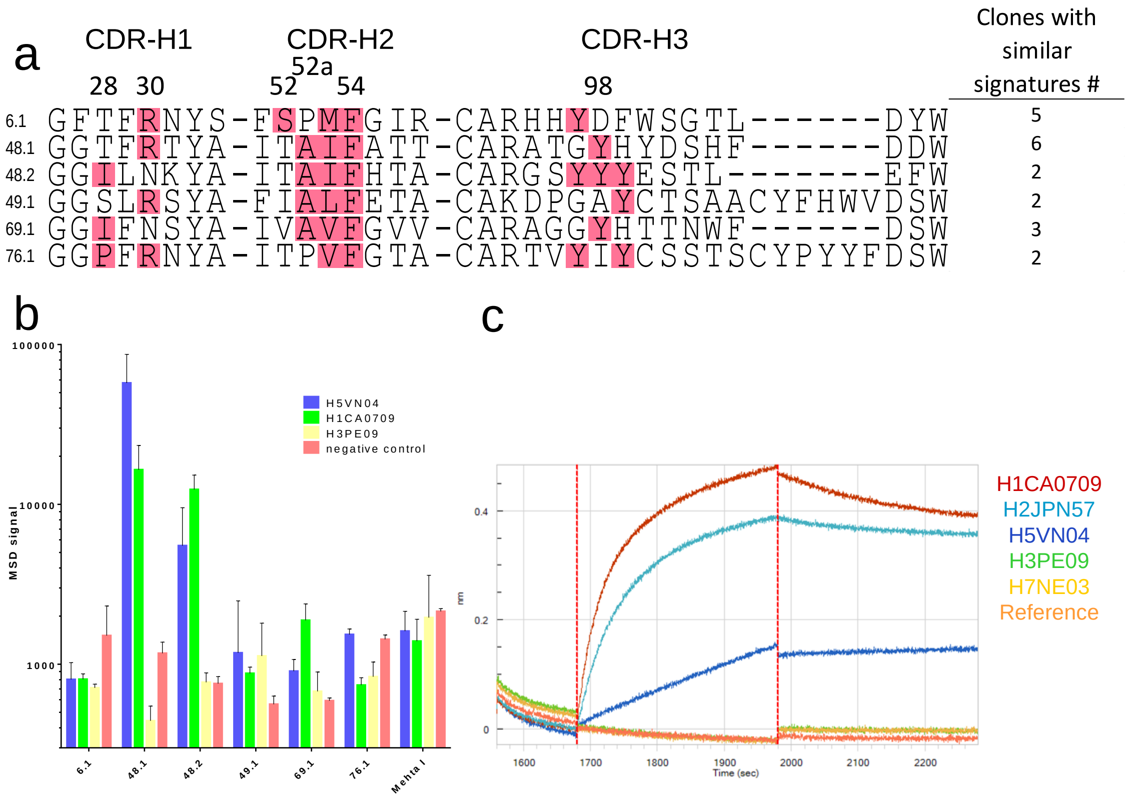

d

KD (M)	KD Error	kon(1/Ms)	kon Error	kdis(1/s)	kdis Error
6.37E-10	9.61E-12	6.53E+05	8.57E+03	4.16E-04	3.11E-06

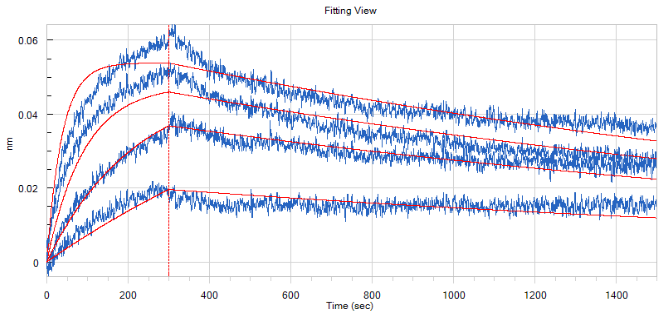

Supplementary Figure 5. The binding activities of synthesized HV1-69-sBnAb precursor clones to hemagglutinins.

(**a**) Six clones characterized by notable HV1-69-sBnAb signatures (maroon residues) were cloned into phagemid light chain shuffle libraries (kappa/lambda). For each clone, also shown is the number of clonally related or duplicated clones (**b**) The 1:1 mixed lambda and kappa phagemid libraries were selected against H5VN04 and the rescued bulk phagemid libraries were analyzed for binding activities against H5VN04, H1CA0709, H3PE09, and against a negative control protein, using 1e13 phagemid particles/mL. Mehta I was used as a control phagemid library. (**c**) Binding kinetic profile of clone 48.1 as scFv-Fc against 10 µg/ml of H1CA0709, H2JPN57, H5VN04, H3PE09, and H7NE03. (**d**) Detailed binding kinetics of clone 48.1 against H2JPN57.

Supplementary Figure 5 text. Synthesizing putative HV1-69-sBnAbs directly from the NGS repertoire data.

We mined the expressed Ab repertoire data for VH genes that are defined by CDR-H3 Tyr 98±1, CDR-H2 Phe54 and by both HV1-69-sBnAb distinctive CDR-H1 and CDR-H2 substitutions. Of the total ~100,000 *IGHV1-69* clones that were analyzed, 20 VH genes met these criteria, and included hydrophobic residue at position 53. Five VH genes, for which duplicate or related clones were identified, were synthesized including two clones (48.1 and 49.1) that contained the dual CDR-H1/H2 substitutions of Ala52 and Arg30 that we[2] and others[8,9] have recently identified, along with 3 other VH genes that had different dual CDR-H1/H2 V-segment signatures (6.1, 48.2 & 69.1). Additional VH gene (76.1) was chosen from a subset that is defined by a dual CDR-H1 signatures (n = 9) (**Supplementary Fig. 5a**). Since only the rearranged *IGHV1-69* VH segment makes contact with HA, the synthetic VH genes were paired with random kappa and lambda light chain genes to form promiscuous light chain shuffled Ab phagemid libraries[2] and one round of panning was performed against H5VN04.

Supplementary Fig. 5b shows that clones 48.1 48.2 and 69.1 showed heterosubtypic binding activity against H1CA0709. The most potent clone, 48.1 was expressed as a scFv-Fc and heterosubtypic binding activity was performed (**Supplementary Fig. 5c**). In addition, binding kinetics was performed against group 1 influenza A strain H2JPN57 that is out of human circulation. **Supplementary Fig. 5d** shows that 48.1-scFv-Fc exhibited strong kinetic binding properties as defined by the KD value of 6.37×10^{-10} M. Interestingly, the 48.1 and 48.2 clones are derived from the IgG dataset of F/F individual 48 who had high HAI and MN post-vaccination titers as well as high HA stem binding activity immediately and longitudinally post-H5N1 vaccination (**Supplementary Fig. 3**).

Figure S6

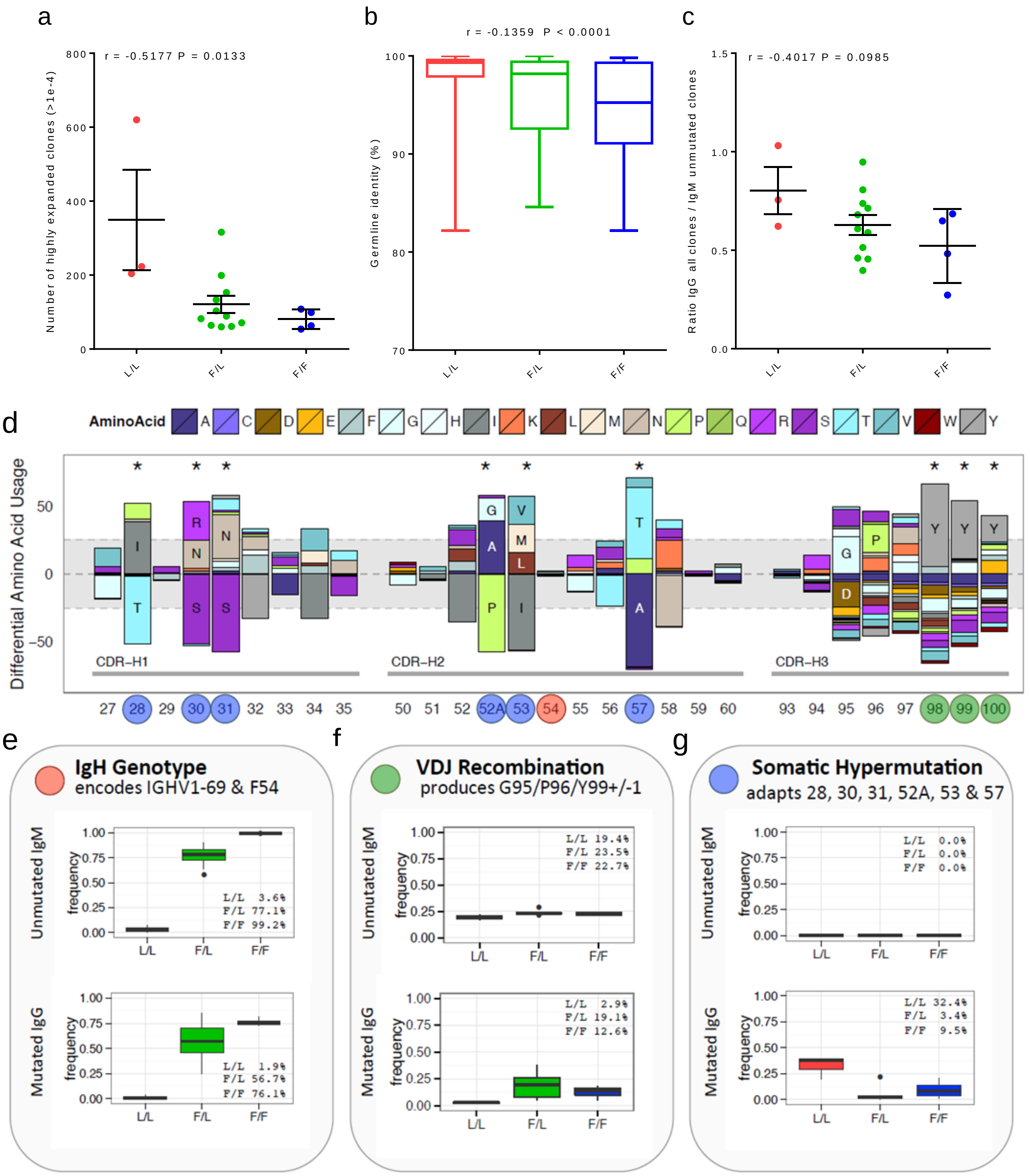

a
r = -0.5177 P = 0.0133
Number of highly expanded clones (>1e-4)
L/L
F/L
F/F
b
r = -0.1359 P < 0.0001
Germline identity (%)
c
r = -0.4017 P = 0.0985
Ratio IgG all clones / IgM unmutated clones
d
AminoAcid
A C D E F G H I K L M N P Q R S T V W Y
Differential Amino Acid Usage
CDR–H1
CDR–H2
CDR–H3
27 28 29 30 31 32 33 34 35 50 51 52 52A 53 54 55 56 57 58 59 60 93 94 95 96 97 98 99 100
e
IgH Genotype
encodes IGHV1-69 & F54
Unmutated IgM
Mutated IgG
frequency
L/L 3.6%
F/L 77.1%
F/F 99.2%
L/L 1.9%
F/L 56.7%
F/F 76.1%
f
VDJ Recombination
produces G95/P96/Y99+/-1
L/L 19.4%
F/L 23.5%
F/F 22.7%
L/L 2.9%
F/L 19.1%
F/F 12.6%
g
Somatic Hypermutation
adapts 28, 30, 31, 52A, 53 & 57
L/L 0.0%
F/L 0.0%
F/F 0.0%
L/L 32.4%
F/L 3.4%
F/F 9.5%

Supplementary Figure 6. Variances in *IGHV1-69* clonal expansion, IgG to IgM ratio, and expansion in-situ evolution of HV1-69-sBnAb precursors.

The three genotypic groups were analyzed for the number of highly expanded clones (frequency of >1e-4) (**a**); their percent germline identity (**b**); and the ratio of IgG to IgM clones defined by unmutated V-segments (**c**). Spearman correlation coefficients (r) were used to summarize the association. Error bars represent standard error of mean; (**d-f**) in order to deconstruct the *in-situ* evolution of HV1-69-sBnAb precursors, the positional amino acid variability of 57 HV1-69-sBnAbs were compared to the total *IGHV1-69* repertoires of F/F individuals. (**d**) Numerous amino acids in the rearranged VH segment are over-represented in the HV1-69-sBnAbs. Positions under significant selection are indicated by asterisks. HV1-69-sBnAb precursor signature variation can be classified into genotype derived allele variation, VDJ recombination-derived CDR-H3 variation, and somatic hypermutation-derived positional arming (highlighted as red (**e**), green (**f)** and blue (**g**), respectively). (**e**) IGH genotype dictates the abundance of total *IGHV1-69* and *IGHV1-69*+F54 BCRs in the repertoire. In both unmutated IgM and class-switched IgG, F54 *IGHV1-69* receptors are abundant in F-allele bearing individuals and nearly absent in L/L homozygotes. (**f**) Generation of HV1-69-sBnAbs CDR3 signatures, dominated by Tyr98-100, a preference for Gly95 and Pro96, and an aversion to Asp95 though VDJ recombination. In the unmutated IgM repertoire, all genotypes have similar frequencies of progenitor HV1-69-sBnAbs CDR-H3 signatures, but only remain elevated in class-switched IgG memory in individuals with F/L or F/F Phe54 genotypes, while loss of HV1-69-sBnAbs CDR-H3 in memory is L-recessive. (**g**) V-segment somatic hypermutation favors six specific positional amino acid changes in HV1-69-sBnAbs. These substitutions are absent in the unmutated IgM, but are expanded in IgG memory. L-allele homozygotes exhibit elevated SHM at these sites.

Figure S7

a

F/L SNP*	SNP2	ASN r^2	ASN genotype counts	EUR r^2	EUR genotype counts	AFR r^2	AFR genotype counts	SNP2, CHR:BP	Location
rs55891010	rs55891010	NA	126(F/F)/266(F/L)/112(L/L)	NA	196(F/F)/237(F/L)/70(L/L)	NA	236(F/F)/411(F/L)/14(L/L)	14:107170062	*IGHV1-69*, coding
rs55891010	rs11845244	0.731	131/267/106	0.894	207/227/69	0.018	259/386/16	14:107170077	*IGHV1-69*, coding
rs55891010	rs2187989	0.804	126/248/130	0.732	182/238/83	0.006	2/63/596	14:107170219	*IGHV1-69*, coding
rs55891010	rs10220412	0.843	122/253/129	0.767	179/236/88	0.131	34/379/248	14:107170410	*IGHV1-69*, 5' UTR
rs55891010	rs11623124	0.836	130/253/121	0.514	91/351/61	0.076	338/309/14	14:107170524	Upstream of *IGHV1-69*

*This SNP encodes the phenylalanine/leucine variant in IGHV1-69, amino acid position 54.

b

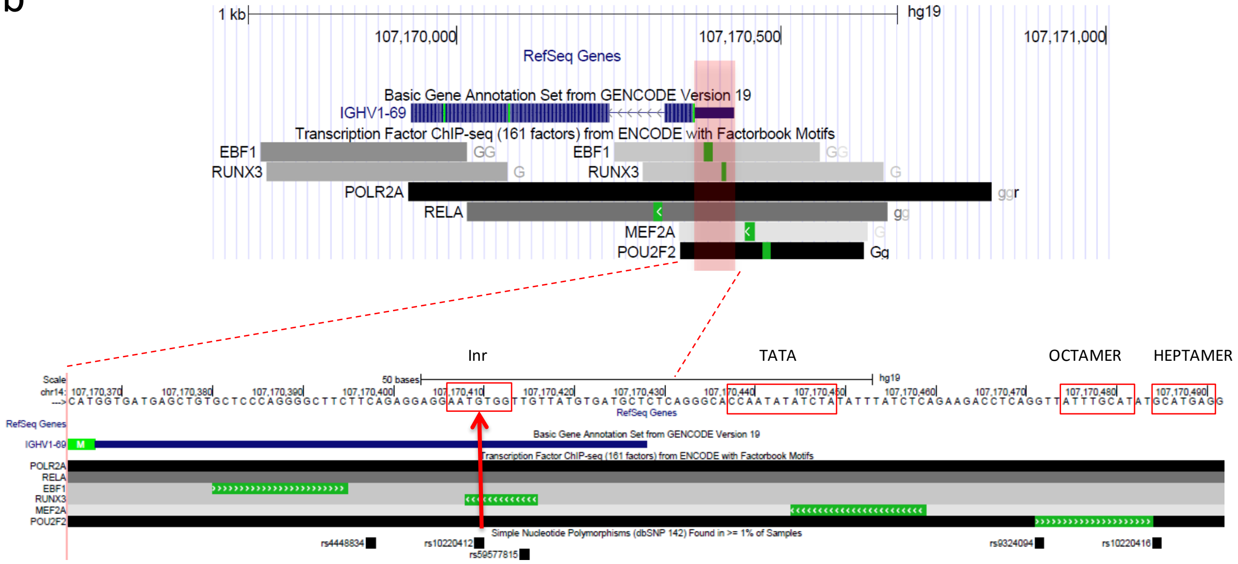

Supplementary Figure 7. Assessment of linkage disequilibrium between the *IGHV1-69* F/L polymorphism (rs55891010) and single nucleotide polymorphisms in the vicinity of *IGHV1-69.*

SNPs within the vicinity of *IGHV1-69* (+- 1.5 kb; GRCh37, chr14:107168431-107171928) that exhibited strong linkage disequilibrium (LD) with the F/L variant (rs55891010), using data from the 1KG phase3 dataset for African (n = 661), Asian (n = 504), and European (n = 503) populations and as assessed using vcftool[10] were identified. (**a**) Only four SNPs had an $r^2 > 0.8$ in at least one of the three populations; two of the identified SNPs represented additional coding variants within *IGHV1-69* and the remaining two occurred upstream of the leader sequence ATG start codon. (**b**) The SNP rs10220412 was found to reside in the 5' UTR of *IGHV1-69* and within an annotated binding motif of the B cell associated protein RUNX3, which has been shown to bind to this region in a lymphoblastoid cell line ChIP-seq dataset[11]. This SNP also resides within the promoter initiator element[12]. (**Upper panel**), image of the genomic region analyzed including annotations of *IGHV1-69,* and binding coordinates of transcription factors determined by ChIP-seq. (**Lower panel**), details of the region surrounding rs10220412 (red arrow), which was shown to be in LD with rs55891010 in the Asian and European populations. The 5'UTR region is shown as a horizontal blue bar and the transcription factor sites of initiator element (Inr), TATA, octamer and heptamer are annotated with red empty boxes. rs10220412 is shown to reside in both the Inr site and in the core consensus motif of RUNX3 (green box), which was identified to bind to this position in a ChIP-seq dataset generated from a lymphoblastoid cell line from the 1000 Genomes Project individual, NA12878. This individual is of European ancestry and has an *IGHV1-69* F/L genotype.

Figure S8

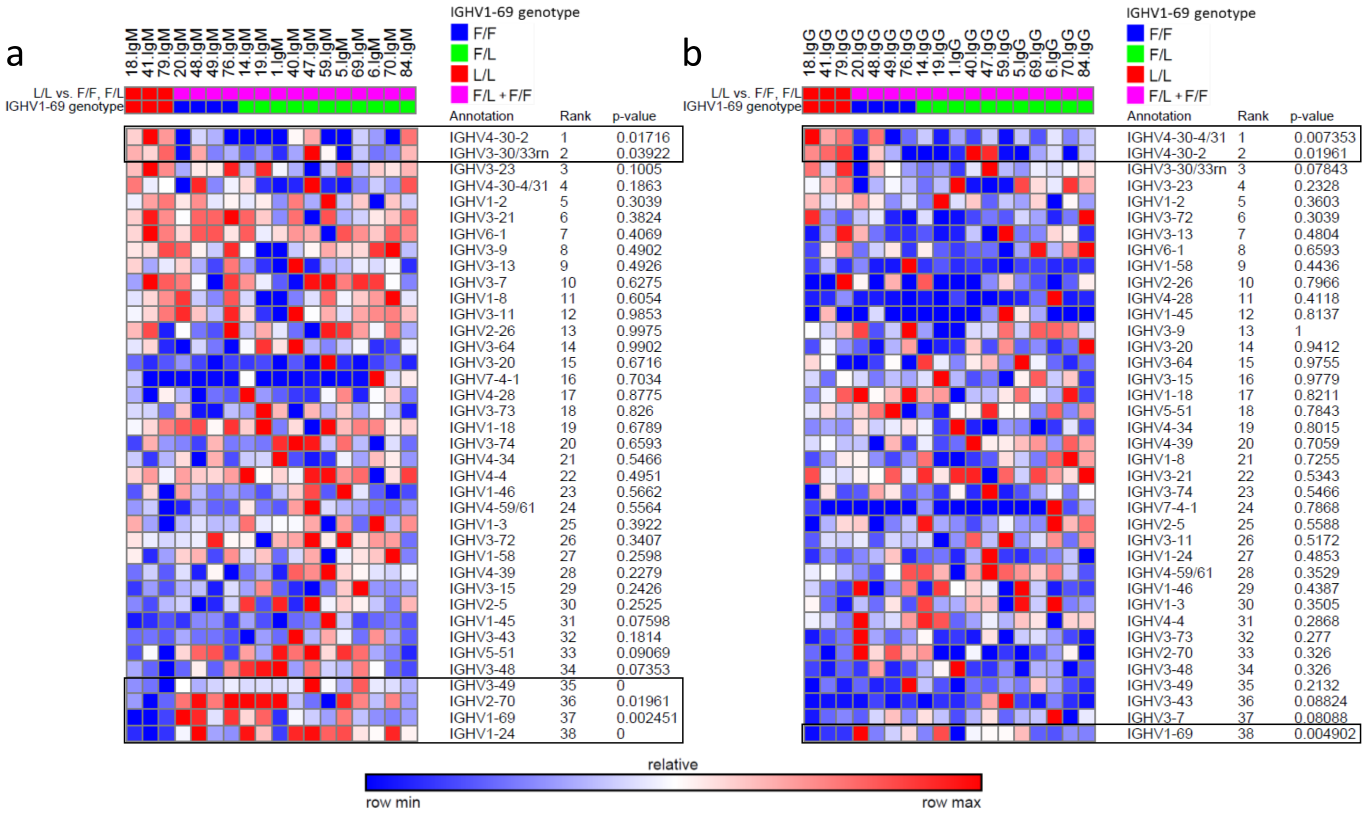

Supplementary Figure 8. Comparing V-gene usages between the L/L group and a combined F/L and F/F group using Heatmaps.

The GENE-E program (Joshua Gould, Broad Institute) was used for generating heatmaps from the V-genes frequencies tabulated in **Fig. 4** the IgM subset characterized by unmutated V-segments (**a**) and the IgG subset (**b**). The numbers on the top of the heatmaps designate sample names of the 18 individuals and square colors refer to their F/L genotype. Relative coloring scheme was chosen, with row minimum colored solid blue and row maximum colored solid red. The marker selection tool was used for comparing the L/L group with the combined F/L-F/F group (compare red squares to purple squares). The V-genes are ordered based on the difference in their utilization between the two groups. On the top of the Table are V-genes that are overrepresented in the L/L group and on the bottom are underrepresented V-genes. V-genes within black rectangles are those with $P < 0.05$.

Figure S9

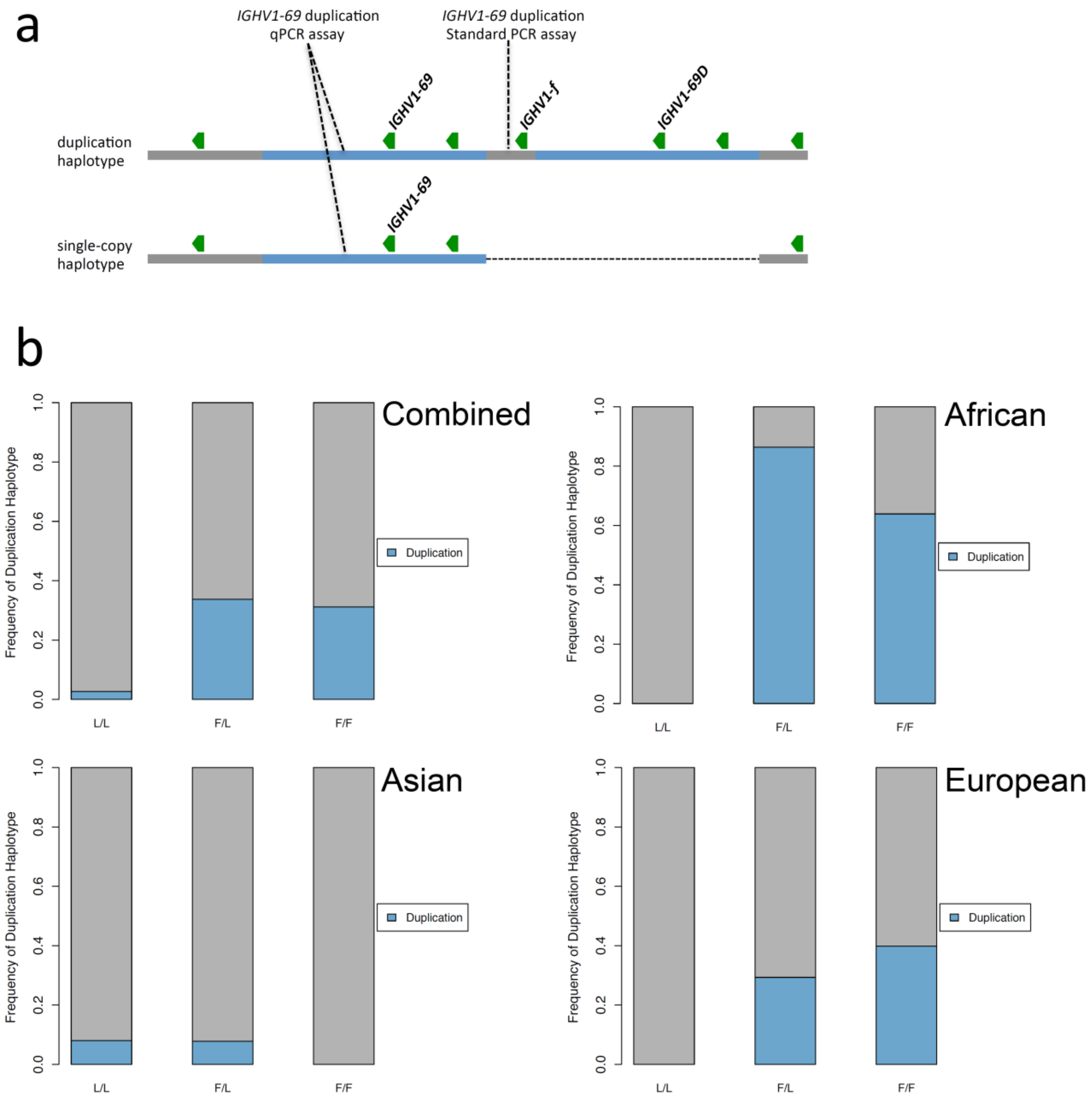
a
IGHV1-69 duplication qPCR assay
IGHV1-69 duplication Standard PCR assay
IGHV1-69
IGHV1-f
IGHV1-69D
duplication haplotype
single-copy haplotype
b
Combined
African
Asian
European
Frequency of Duplication Haplotype
Duplication
L/L
F/L
F/F
0.0
0.2
0.4
0.6
0.8
1.0

Supplementary Figure 9. Population-specific associations between *IGHV1-69* F/L genotype and germline copy number.

(**a**) Schematic of the *IGHV1-69* region duplication structure, including the locations of qPCR and standard PCR assays designed by Watson et al[3] (see citation[3] for assay details), which were previously used to estimate the copy number of *IGHV1-69* and the presence/absence of the duplication haplotype, respectively. To investigate the relationship between *IGHV1-69* copy number and rs55891010 genotype (*i.e.*, L/L, F/L, F/F), we used CNV/duplication genotypes generated in Watson et al[3] with these assays in a subset of samples from three broad ethnic groups (African, n=78; Asian, n=85; European, n=125), for which rs55891010 SNP genotype data were also available from the 1000 Genomes Project. (**b**) Complementing qPCR copy number estimates displayed in main **Fig. 5**, the standard PCR assay targeting sequence specific to the duplication haplotype revealed a similar relationship between *IGHV1-69* duplication frequency and rs55891010 genotype; estimated duplication haplotype frequencies were calculated based on the fraction of single-copy haplotype homozygotes, assuming Hardy-Weinberg equilibrium. Consistent with copy number estimates in main **Fig. 5**, the frequency of the duplication haplotype varies considerably between populations and rs55891010 genotype, with the highest overall frequencies observed in Africans, and the lowest in Asians.

Figure S10

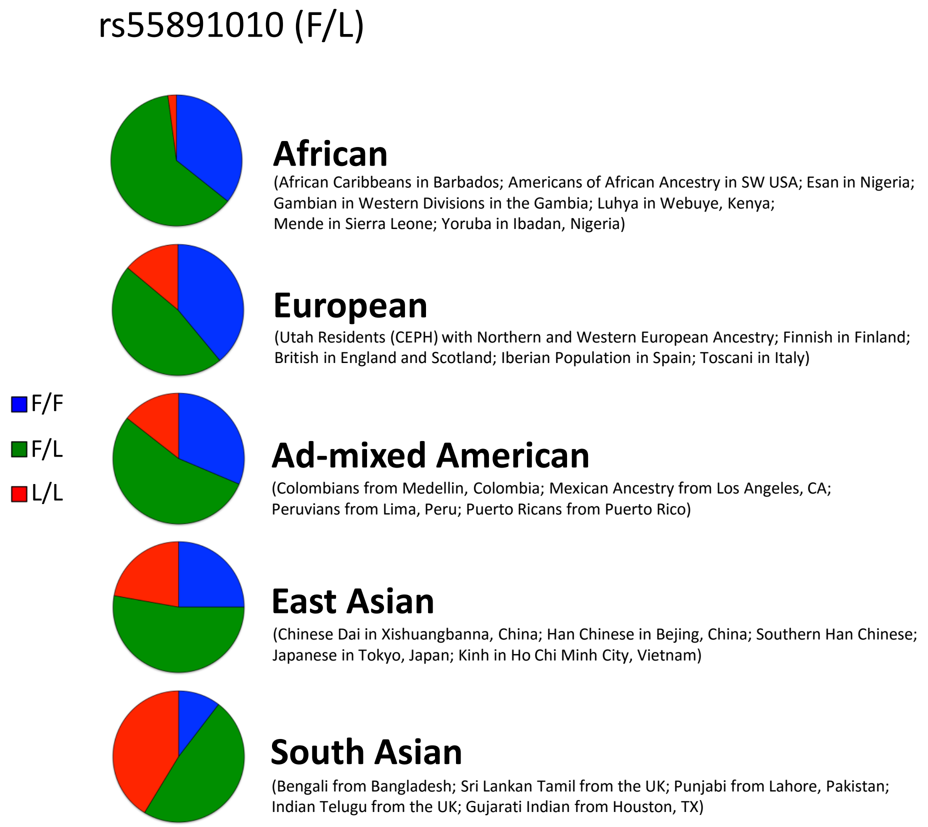

Supplementary Figure 10. *IGHV1-69* F/L polymorphism genotype frequencies in five broad human ethnicities.

Genotype frequencies at the *IGHV1-69* F/L polymorphism (SNP ID: rs55891010) in African, European, Ad-mixed American, East Asian, and South Asian populations, based on genotypes downloaded from the Phase3 1000 Genomes Project dataset (1000 Genomes Project Consortium, 2010)[13]. Genotypes shown are encoded as F/F, F/L (L/F or F/L), and L/L, corresponding to A/A, G/A, A/G and G/G SNP genotypes, respectively. Extreme variations in genotype frequencies are shown across the human populations, with L/L frequency as high as 41% in South Asians, and as low as ~2% in the African population. Subpopulations comprising each broader ethnic group are indicated in parentheses.

Figure S11

Fused IGHV genes	Organization on the IGH locus (telomeric to centromeric)		The order of IGHV genes in Fig. 4
	IGHV1-69		*IGHV1-69*
IGHV3-53/66	*IGHV3-66*		not included
	IGHV3-64		*IGHV3-64*
IGHV4-59/61	*IGHV4-61*		*IGHV4-59/61*
	IGHV4-59		
	IGHV1-58		*IGHV1-58*
	IGHV3-53		not included
	IGHV5-51		*IGHV5-51*
	IGHV4-34	*IGHV3-30-5*	*IGHV4-34*
IGHV3-30/33rn	*IGHV3-33*	*IGHV4-30-4*	IGHV4-30-4/31
	IGHV4-31	*IGHV3-30-3*	IGHV4-30-2
IGHV4-30-4/31	*IGHV3-30*	*IGHV4-30-2*	IGHV3-30/33rn
	IGHV4-28	*IGHV4-30-1*	IGHV4-28

Supplementary Figure 11. The positions of highly related IGHV genes that cannot be distinguished with confidence by the antibodyome pipeline.

The antibodyome pipeline used in this study is able to distinguish 63 IGHV germline genes, which include functional genes, open reading frame genes and pseudogenes. In order to avoid classification error, a subset of very similar V-genes are presented in a combined notation. In the functional germline gene group, these are *IGHV3-53/66*, *IGHV4-59/61*, *IGHV3-30/33* and *IGHV4-30-4/31*. In the middle column of the Table the IGHV pair of each combined germline gene is shown with respect to its IGH locus position. In the right hand column the order used in **Fig. 4** is detailed. Since the purpose of **Fig. 4** is to assess correlations between *IGHV1-69* genotype and utilization of other V-genes in respect to their localization in the IGH locus we decided not to include *IGHV3-53/66* as these two genes are separated by a large genomic distance.

References

1 Lefranc, M. P. Immunoglobulins: 25 years of immunoinformatics and IMGT-ONTOLOGY. *Biomolecules* **4**, 1102-1139, doi:10.3390/biom4041102 (2014).

2 Avnir, Y. *et al.* Molecular signatures of hemagglutinin stem-directed heterosubtypic human neutralizing antibodies against influenza A viruses. *PLoS pathogens* **10**, e1004103, doi:10.1371/journal.ppat.1004103 (2014).

3 Watson, C. T. *et al.* Complete haplotype sequence of the human immunoglobulin heavy-chain variable, diversity, and joining genes and characterization of allelic and copy-number variation. *American journal of human genetics* **92**, 530-546, doi:10.1016/j.ajhg.2013.03.004 (2013).

4 Sasso, E. H., Willems van Dijk, K., Bull, A. P. & Milner, E. C. A fetally expressed immunoglobulin VH1 gene belongs to a complex set of alleles. *The Journal of clinical investigation* **91**, 2358-2367, doi:10.1172/JCI116468 (1993).

5 Milner, E. C., Hufnagle, W. O., Glas, A. M., Suzuki, I. & Alexander, C. Polymorphism and utilization of human VH Genes. *Annals of the New York Academy of Sciences* **764**, 50-61 (1995).

6 Sui, J. *et al.* Structural and functional bases for broad-spectrum neutralization of avian and human influenza A viruses. *Nature structural & molecular biology* **16**, 265-273, doi:10.1038/nsmb.1566 (2009).

7 Lingwood, D. *et al.* Structural and genetic basis for development of broadly neutralizing influenza antibodies. *Nature* **489**, 566-570, doi:10.1038/nature11371 (2012).

8 Pappas, L. *et al.* Rapid development of broadly influenza neutralizing antibodies through redundant mutations. *Nature* **516**, 418-422, doi:10.1038/nature13764 (2014).

9 Whittle, J. R. *et al.* Flow cytometry reveals that H5N1 vaccination elicits cross-reactive stem-directed antibodies from multiple Ig heavy-chain lineages. *Journal of virology* **88**, 4047-4057, doi:10.1128/JVI.03422-13 (2014).

10 Danecek, P. *et al.* The variant call format and VCFtools. *Bioinformatics* **27**, 2156-2158, doi:10.1093/bioinformatics/btr330 (2011).

11 Harrow, J. *et al.* GENCODE: the reference human genome annotation for The ENCODE Project. *Genome research* **22**, 1760-1774, doi:10.1101/gr.135350.111 (2012).

12 Roy, A. L., Sen, R. & Roeder, R. G. Enhancer-promoter communication and transcriptional regulation of Igh. *Trends in immunology* **32**, 532-539, doi:10.1016/j.it.2011.06.012 (2011).

13 Abecasis, G. R. *et al.* A map of human genome variation from population-scale sequencing. *Nature* **467**, 1061-1073, doi:10.1038/nature09534 (2010).
